# Characterization and expression analysis of the *WRKY* gene family in moso bamboo

**DOI:** 10.1038/s41598-017-06701-2

**Published:** 2017-07-27

**Authors:** Long Li, Shaohua Mu, Zhanchao Cheng, Yuanwen Cheng, Ying Zhang, Ying Miao, Chenglin Hou, Xueping Li, Jian Gao

**Affiliations:** 1 International Center for Bamboo and Rattan, Key Laboratory of Bamboo and Rattan Science and Technology, State Forestry Administration, Beijing, 100102 People’s Republic of China; 2 College of Forestry, Northwest Agriculture & Forestry University, Yangling, Shaanxi 712100 People’s Republic of China; 3 Center for Molecular Cell and Systems Biology, College of Life Sciences, Fujian Agriculture and Forestry University, Fuzhou, 350002 People’s Republic of China; 4 Department of Microbiology, College of Life Science, Capital Normal University, Beijing, 100048 People’s Republic of China

## Abstract

The WRKY family of transcription factors (TFs) is one of the ten largest families of TFs in higher plants and has been implicated in multiple biological processes. Here, we identified 121 WRKY TFs in moso bamboo, including five novel members that were not annotated in the *Phyllostachys edulis* genomic database. Estimation of the divergence time of paralogous gene pairs revealed an important role of the recent whole-genome duplication in the expansion of the WRKY family. Expression analysis based on quantitative reverse-transcription polymerase chain reaction (qRT-PCR) data revealed that a large number of *PheWRKY* genes varied significantly under cold or drought stress treatments, which could be defined as abiotic stress-responsive genes. The overexpression of *PheWRKY72-2* in *Arabidopsis* resulted in a decreased sensitivity to drought stress during early seedling growth. *PheWRKY72-2* may enhance plant tolerance to stress by functioning as a positive regulator of stoma closure. Our study provides a theoretical foundation and some experimental evidence for further functional verification of the PheWRKY family of TFs.

## Introduction

Moso bamboo (*Phyllostachys edulis*) is a large, woody bamboo with the highest ecological, economic, and cultural value of all bamboo types and accounts for 70% of the total cultivated bamboo worldwide^1^. Moso bamboo belongs to the monophyletic Bambusoideae, Ehrhartoideae, Pooideae (BEP) clade of the grass family (Poaceae). Moso bamboo is a perennial plant characterized by its woody culm and a prolonged vegetative phase that lasts for decades before flowering^1^. It is one of the most important non-timber forest resources in south-eastern China because of its striking shoot growth rate. Indeed, the shoot can grow as long as 1 m within 24 hrs and reaches a final height of 5–20 m in 45 to 60 days^2^. Interestingly, multiple moso bamboo plants often flower and die after flowering simultaneously^1^. Moreover, the plants rarely flower in a natural setting. Transcriptomic studies in *P. edulis* provide an excellent opportunity to investigate genes that could be useful for improving plant growth^3^. RNA sequencing studies suggest that drought stress may be related to flowering in bamboo^4^. *P. edulis* has a tetraploid origin that underwent whole-genome duplication approximately 7–12 million years ago (mya). Collinearity analysis of *Oryza sativa* and bamboo revealed that a large number of genes were lost at the same time^4^.

The WRKY family is one of the ten largest transcription factor (TF) families in higher plants^5^. The first cDNA encoding a WRKY protein to be cloned was *SPF1* from *Pachyrhizus erosus* ^6^. Since then, several WRKY family member proteins have been identified from various plant species^7–11^, all of which play key roles in the response to ever-changing internal and external stimuli. The WRKY TFs are named after the WRKY domain, which consists of approximately 60 amino acids. These TFs play vital regulatory roles in developmental and physiological processes, such as seed dormancy^12^, embryo morphogenesis^13^, plant growth^14^, senescence^15, 16^, and metabolism^17, 18^. Furthermore, WRKY TFs are involved in responses to various abiotic and biotic stresses, such as bacteria^19, 20^, fungi^15, 21^, nematodes^22^, wounding^23^, heat, drought, salinity and cold^24^. Most WRKY family members contain highly conserved heptapeptides (WRKYGQK) at their N-terminals and a C_2_H_2_ or C_2_HC zinc-finger motif at their C-terminals^7, 23, 25^. However, some have altered motifs, including WRKYGEK and WRKYGKK^21, 26^. WRKY proteins are categorized into three distinct groups based on both the number of WRKY domains and the features of their zinc-finger-like motifs. The WRKY TFs with two WRKY domains containing C_2_H_2_ zinc-finger motifs belong to group 1. Group 2 TFs have a single WRKY domain that includes a C_2_H_2_ zinc-finger motif and are further divided into five subgroups based on their phylogenetic relationships. Group 3 TFs contain a single WRKY domain with a C_2_HC zinc-finger motif^27^.

Gao *et al*. (2013) identified a *P. edulis* WRKY family gene, *PheWRKY67-2* (GenBank accession number: FP101056.1), involved in the defence of *P. edulis* against the pathogens *Ceratosphaeria phyllostachydis* and *Arthrinium phaeospermum* ^28^. However, to date, no studies have addressed the WRKY gene family and its function in the organ growth and development of *P. edulis*. Here, for the first time, we performed a detailed analysis of the subgroup classification, gene structure and conserved motif composition of 121 WRKY TFs in the *P. edulis* genome. We analysed the expression patterns of *PheWRKYs* from floral expression profiles and fast-growing shoot expression profiles to determine whether the expression patterns of *PheWRKYs* were related to fast-growing shoots and flower development in *P. edulis*. We further studied changes in expression of the *PheWRKY* genes in response to abiotic stress (drought or cold) in *P. edulis* seedlings. Furthermore, a drought stress and cold stress-inducible gene, *PheWRKY72-2*, was characterized in transgenic *Arabidopsis* to comprehensively understand its role in the response to abiotic stress. Our study provided a theoretical foundation and some experimental evidence for further functional verification of PheWRKYs.

## Results

### Identification of the WRKY family of TFs

We identified 121 WRKY members in the *P. edulis* genomic database (alternative splicing variants were not considered; Table 1), including five novel members that were not annotated in the *P. edulis* genomic database (Table S1). Because all the proteins and CDS sequences obtained from moso bamboo genome database were annotated by their putative orthologous sequences of *O. sativa*, so we renamed each moso bamboo WRKY sequences based on the individual similarity with rice WRKY proteins by blast method, and the different proteins of moso bamboo which corresponded to the same sequences in rice were suffixed with ‘−1’, ‘−2’, ‘−3’, ‘−4’ depending on levels of sequences similarity (Table S2)^29^. The polypeptides encoded by the *PheWRKY* genes ranged from 159 to 1,168 amino acids in length, with predicted pI values ranging from 4.7 to 10.1 and molecular weights ranging from 19.77 to 131.06 kD (Table S3).

**Table 1:** The WRKY genes in *P. edulis*.

ID	WRKY group	Zinc-finger type			
		Conserved heptapeptide	Zinc-finger type	m	n
PheWRKY1-1	group2b	WRKYGQK	C2H2	5	23
PheWRKY1-2	group2b	WRKYGQK	C2H2	5	23
PheWRKY2	group2e	WRKYGQK	C2H2	5	23
PheWRKY3-1	group2c	WRKYGQK	C2H2	4	23
PheWRKY3-2	group2c	WRKYGQK	C2H2	4	23
PheWRKY4	group1	WRKYGQK/WRKYGQK	C2H2	4/4	22/23
PheWRKY5-1	group2b	WRKYGQK	C2H2	5	23
PheWRKY5-2	/	WRKYGQK	/	/	/
PheWRKY7	group2c	WRKYGKK	C2H2	4	23
PheWRKY8	group2c	WRKYGQK	C2H2	4	23
PheWRKY9-1	group2b	WRKYGQK	C2H2	5	88
PheWRKY9-2	group2b	WRKYGQK	C2H2	5	23
PheWRKY10	group2c	WRKYGQK	C2H2	4	23
PheWRKY11-1	group2c	WRKYGQK	C2H2	4	23
PheWRKY11-2	group2c	WRKYGQK	C2H2	4	23
PheWRKY11-3	group2c	WKKYGQK	C2H2	4	24
PheWRKY12	group2e	WRKYGQK	C2H2	5	23
PheWRKY13-1	group2e	WRKYGQK	C2H2	5	23
PheWRKY13-2	group2e	WRKYGQK	C2H2	5	23
PheWRKY13-3	group2e	WRKYGQK	C2H2	5	23
PheWRKY14	group2e	WRKYGQK	C2H2	5	23
PheWRKY15-1	group3	WRKYGQK	C2HC	7	23
PheWRKY15-2	group3	WRKYGQK	C2HC	7	23
PheWRKY16	group2c	WRKYGQK	C2H2	4	23
PheWRKY17-1	group2c	WRKYGQK	C2H2	4	23
PheWRKY17-2	group2c	WRKYGQK	C2H2	4	23
PheWRKY17-3	/	WRKYGQK	/	/	/
PheWRKY19-1	group3	WRKYGQK	C2HC	7	23
PheWRKY19-2	group3	WRKYGQK	C2HC	7	23
PheWRKY19-3	group3	WRKYGQK	C2HC	7	23
PheWRKY21	group3	WRKYGQK	C2HC	6	23
PheWRKY22-1	group3	WRKYGQK	C2HC	7	24
PheWRKY22-2	group3	WRKYGQK	C2HC	7	24
PheWRKY24-1	group1	WRKYGQK/WRKYGQK	C2H2	4/4	22/23
PheWRKY24-2	group1	WRKYGQK/WRKYGQK	C2H2	4/4	22/23
PheWRKY25	/	/	C2H2	4	23
PheWRKY26-1	group2c	WRKYGKK	C2H2	4	23
PheWRKY26-2	group2c	WRKYGKK	C2H2	4	23
PheWRKY28	group2a	WRKYGQK	C2H2	5	23
PheWRKY29-1	group2c	WRKYGQK	C2H2	4	23
PheWRKY29-2	group2c	WRKYGQK	C2H2	4	23
PheWRKY29-3	group2c	WRKYGQK	C2H2	4	23
PheWRKY29-4	group2c	WRKYGQK	C2H2	4	23
PheWRKY34-1	/	WRKYGQK	/	/	/
PheWRKY34-2	group2c	WRKYGQK	C2H2	4	23
PheWRKY35-1	group1	WRKYGQK/WRKYGQK	C2H2	4/4	22/23
PheWRKY35-2*	group1	WRKYGQK/WRKYGQK	C2H2	4	22
PheWRKY36	group2c	WRKYGQK	C2H2	4	23
PheWRKY39-1	group2e	WRKYGQK	C2H2	5	23
PheWRKY39-2	group2e	WRKYGQK	C2H2	5	23
PheWRKY39-3	group2e	WRKYGQK	C2H2	5	23
PheWRKY42	group2d	WRKYGQK	C2H2	5	23
PheWRKY43	group2b	WRKYGQK	C2H2	5	23
PheWRKY44	group3	WRKYGQK	C2HC	7	23
PheWRKY45-1	group3	WRKYGQK	C2HC	7	23
PheWRKY45-2	/	/	C2HC	7	23
PheWRKY46-1	group3	WRKYGEK	C2HC	7	23
PheWRKY46-2	group3	WRKYGEK	C2HC	7	24
PheWRKY48-1	group3	WRKYGQK	C2HC	7	23
PheWRKY48-2	group3	WRKYGQK	C2H2	5	23
PheWRKY49	group2c	WRKYGQK	C2H2	4	23
PheWRKY51-2	group2d	WRKYGQK	C2H2	5	23
PheWRKY51-1	group2d	WRKYGQK	C2H2	5	23
PheWRKY65-1	group3	WRKYGQK	C2HC	7	29
PheWRKY65-2	group3	WRKYGQK	C2HC	7	30
PheWRKY53-1	group1	WRKYGQK/WRKYGQK	C2H2	4/4	23/23
PheWRKY53-2	group1	WRKYGQK/WRKYGQK	C2H2	4/4	23/23
PheWRKY55	group3	WRKYGEK	C2HC	7	24
PheWRKY62	group2a	WRKYGQK	C2H2	5	23
PheWRKY66	group2e	WRKYGQK	C2H2	5	23
PheWRKY67-1	group2c	WRKYGKK	C2H2	4	23
PheWRKY67-2	group2c	WRKYGKK	C2H2	4	23
PheWRKY68-1	group2d	WRKYGQK	C2H2	5	23
PheWRKY68-2	/	WRKYGQK	/	/	/
PheWRKY69-1	group3	WRKYGQK	C2HC	7	23
PheWRKY69-2	group3	WRKYGQK	C2HC	7	23
PheWRKY70-1	group1	WRKYGQK/WRKYGQK	C2H2	4/4	22/23
PheWRKY70-2	group1	WRKYGQK/WRKYGQK	C2H2	4/4	22/23
PheWRKY71-1	group2a	WRKYGQK	C2H2	5	23
PheWRKY71-2	group2a	WRKYGQK	C2H2	5	23
PheWRKY72-1	group2c	WRKYGQK	C2H2	4	23
PheWRKY72-2	group2c	WRKYGQK	C2H2	4	23
PheWRKY72-3	group2c	WRKYGQK	C2H2	4	23
PheWRKY73-1	group2b	WRKYGQK	C2H2	5	23
PheWRKY73-2	group2b	WRKYGQK	C2H2	5	23
PheWRKY74-1	group3	WRKYGQK	C2HC	7	23
PheWRKY74-2	group3	WRKYGKK	C2H2	7	23
PheWRKY75	group3	WRKYGQK	C2HC	7	23
PheWRKY76	group2a	WRKYGQK	C2H2	5	23
PheWRKY77-1	group2c	WRKYGQK	C2H2	4	23
PheWRKY77-2	group2c	WRKYGQK	C2H2	4	23
PheWRKY78	group1	WRKYGQK/WRKYGQK	C2H2	4/4	22/23
PheWRKY79-2	/	WRKYGQK	/	/	/
PheWRKY79-1	/	WRKYGQK	/	/	/
PheWRKY80-1	group1	CRKYGQA/WRKYGQK	C2H2	4/4	23/22
PheWRKY80-2	group1	WRKYGQQ/WRKYGQK	C2H2	4/4	22/23
PheWRKY80-3	group1	WRKYGQK/WRKYGQK	C2H2	4/4	22/23
PheWRKY82	group1	WRKYGQK/WRKYGQK	C2H2	4/4	22/23
PheWRKY83-1	group2d	WRKYGQK	C2H2	5	23
PheWRKY83-2	/	WRKYGQK	/	/	/
PheWRKY83-3	group2d	WRKYGQK	C2H2	5	23
PheWRKY85-1*	group1	WRKYGQK/WRKYGQK	C2H2	4	22
PheWRKY85-2	group1	WRKYGQK/WRKYGQK	C2H2	4/4	22/22
PheWRKY88-1	group2e	WRKYGQK	C2H2	5	23
PheWRKY88-2	/	/	C2H2	5	23
PheWRKY89	group2d	WRKYGQK	C2H2	5	23
PheWRKY93	group3	WRKYGQK	C2HC	7	23
PheWRKY95	/	WRKYGQK	/	/	/
PheWRKY96-1*	group1	WRKYGQK/WRKYGQK	C2H2	4	22
PheWRKY96-2*	group1	WRKYGQK/WRKYGQK	C2H2	4	22
PheWRKY96-3*	group1	WRKYGQK/WRKYGQK	C2H2	4	22
PheWRKY97-1	group2b	WRKYGQK	C2H2	5	23
PheWRKY97-2	group2b	WRKYGQK	C2H2	5	23
PheWRKY100	group3	WRKYGQK	C2HC	7	27
PheWRK109	group2b	WRKYGQK	C2H2	4	23
PheWRKY111	group2e	WRKYGQK	C2H2	5	23
PheWRKY114-1	group3	WRKYGEK	C2HC	7	24
PheWRKY114-2	group3	WRKYGEK	C2HC	7	23
PheWRKY116	group3	WRKYGQK	C2HC	7	24
PheWRKY119	group2c	WRKYGQK	C2H2	4	23
PheWRKY125	group1	WRKYGQK/WRKFGQK	C2HC	4/4	23/23

Note: *Indicates the amino acid sequences in group1 have only one integrity WRKY domain and the structure of second WRKY domain delete zinc-finger motif; ‘m’ and ‘n’ refer to the number of residues present in the consensus zinc finger motif C–Xm–C–Xn–HXH/C.

### Structure and classification of PheWRKYs

**Figure 1:**
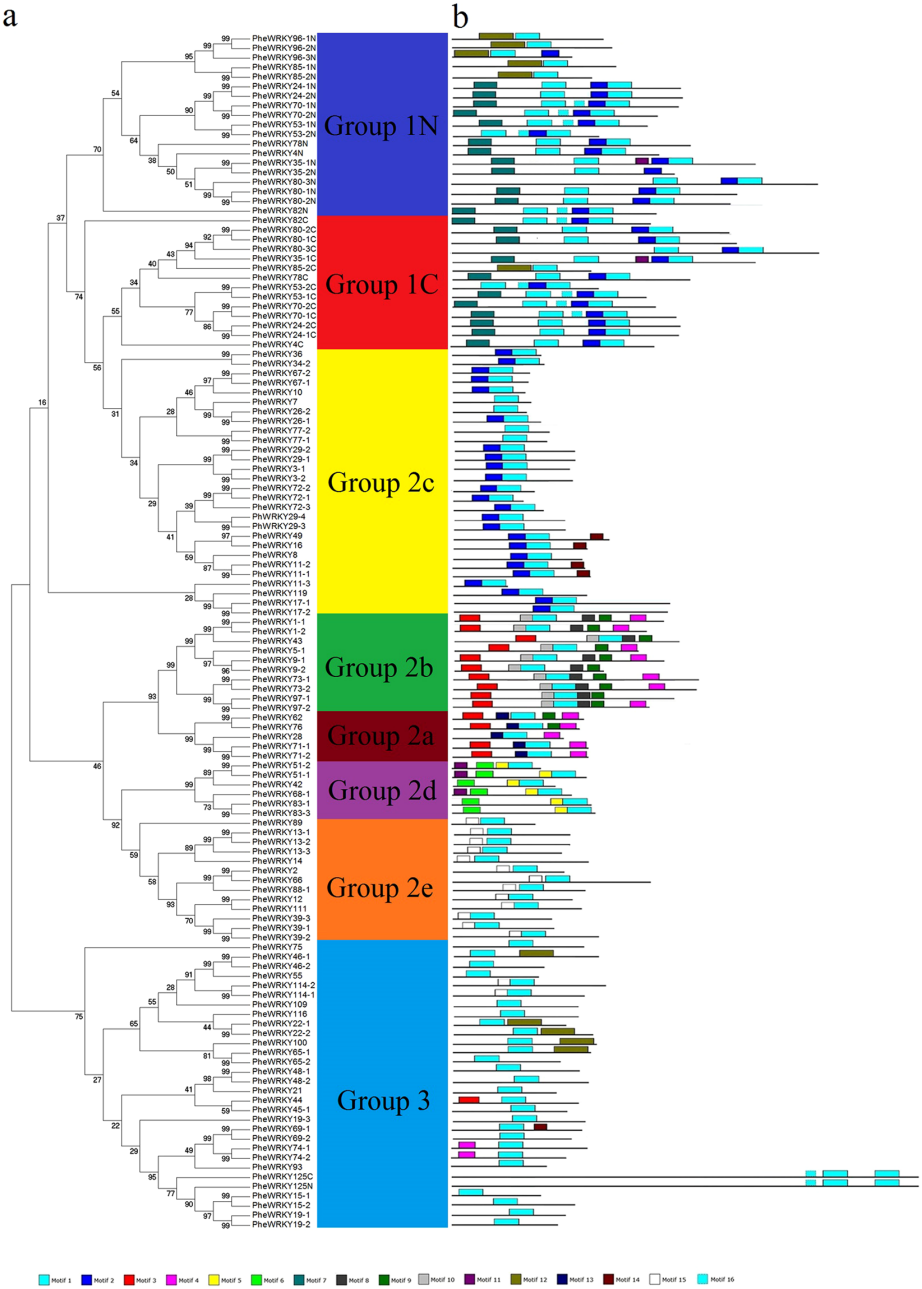
Phylogenetic analysis of the WRKY family of proteins in *P. edulis* **(a)** and the distribution of conserved motifs within each group **(b)**.

According to the zinc-finger structure (Table 1), sequence alignment (Figure S1), conserved motif (Fig. 1 and Figure S2) and gene structure (Figure S3), the PheWRKYs can be classified into three main groups. Twenty PheWRKY proteins that contained two WRKY domains were assigned to group 1. Fifty of these contained two intact WRKY domains. However, the other five members of group 1 (PheWRKY35-2, 85-1, 96-1, 96-2 and 96-3) had only one complete WRKY domain in their N-terminals, and their C-terminal domains lacked a zinc-finger. PheWRKY125, which should have been placed in group 3 because of its C_2_HC type zinc-finger, was assigned to group 1 because it contained two WRKY domains. Except for PheWRKY125, the zinc-finger structures of the PheWRKY proteins in group 1 were of C_2_H_2_ type with a C-X_4_-C-X_22-23_-H-X_1_-H motif (Table 1). Sixty-three PheWRKY proteins had a single WRKY domain, but in 62 members, the motif was C-X_4-5_-C-X_23_-H-X_1_-H; one member had the motif C-X_5_-C-X_88_-H-X_1_-H (PheWRKY9-1). All 62 members were assigned to group 2 and were further classified into five subgroups based on their conserved motif, their gene structure, and phylogenetic analysis. The subgroups included the following: group 2a (5 members), group 2b (11 members), group 2c (28 members), group 2d (7 members), and group 2e (12 members). The zinc-finger structure of group 3 was C_2_HC (27 members), with the motif C-X_6-7_-C-X_23-30_-H-X_1_-C (Table 1). Eleven proteins contained only partial WRKY domains and did not fit in any group. Most group 2c members shared motif 2 with most group 1 protein sequences, which also primarily harboured motifs 7 and 16. Motif 13 appeared only in group 2a, whereas motifs 8, 9 and 10 were found exclusively in group 2b. Group 2d harboured motifs 5, 6 and 11 (Fig. 1b).

Structural analysis of the genes revealed that consisted with previous report^30^, gene family members within the same group shared similar gene structures in terms of their intron numbers and intron phases (Figure S3). The intron phases of most sequences in group 1 were 0 at the first two introns and 2 at the last two introns. Except for *PheWRKY97-1*, the intron phases of all other members from groups 2a and 2b were 0. For groups 2c, 2d, 2e and 3, most introns phases were 2. Group 2b had more introns (average: 4.375) than the other groups. In contrast, almost all members of groups 2a, 2c, 2d, and 2e had only two introns. Generally, the motif and gene structure analysis revealed that members from different species assigned to the same group always shared similar motifs, intron numbers, and phases (Fig. 1b, Figure S3).

Although the WRKYGQK motif was highly conserved, we found several sequence variations in 15 *P. edulis* WRKY proteins, most of which belonged to groups 3 and 2c (Table S4). We identified WRKYGKK as the most common variant in six domains, whereas WRKYGEK was common to five domains. The other four variants—WRKYGKK, CRKYGQA, WRKYGQQ, and WRKFGQK—were found in a single WRKY domain in PheWRKY7-3, PheWRKY80-1 (N-terminal), PheWRKY80-2 (N-terminal), and PheWRKY61 (C-terminal), respectively.

**Figure 2:**
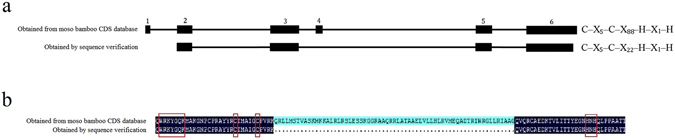
**(a)** Structure of the *PheWRKY9-1* gene. The sequence obtained from the *P. edulis* CDS database is indicated on top, and the sequence obtained by sequencing verification is shown below the sequence. **(b)** Sequence alignment of the PheWRKY9-1 sequence obtained from the *P. edulis* CDS database and the sequence obtained by sequencing verification.

To confirm the specific zinc-finger structure of PheWRKY9-1 (C-X_5_-C-X_88_-H-X_1_-H), we designed a pair of primers at the 5′-UTR and 3′-UTR of *PheWRKY9*-*1*. Different RNA samples isolated from different tissues, including one-year-old leaves, two-year-old leaves, flowers, one-year-old culms, shoots, seeds, roots, and seedlings under various abiotic stress, were used as clone templates. Although we did not obtain the CDS sequence of *PheWRKY9*-*1*, which is annotated in the moso bamboo CDS database, from any of the tissues, we did identify a gene with a normal zinc-finger structure (C-X_5_-C-X_23_-H-X_1_-H). Compared with the annotated *PheWRKY9*-*1* sequence from the moso bamboo CDS database, this gene lacked two exons (Fig. 2a and b), as revealed by sequencing studies. In addition, whether the specific zinc-finger exists or is merely a mis-annotation could not be determined.

### Phylogenetic relationships

**Figure 3:**
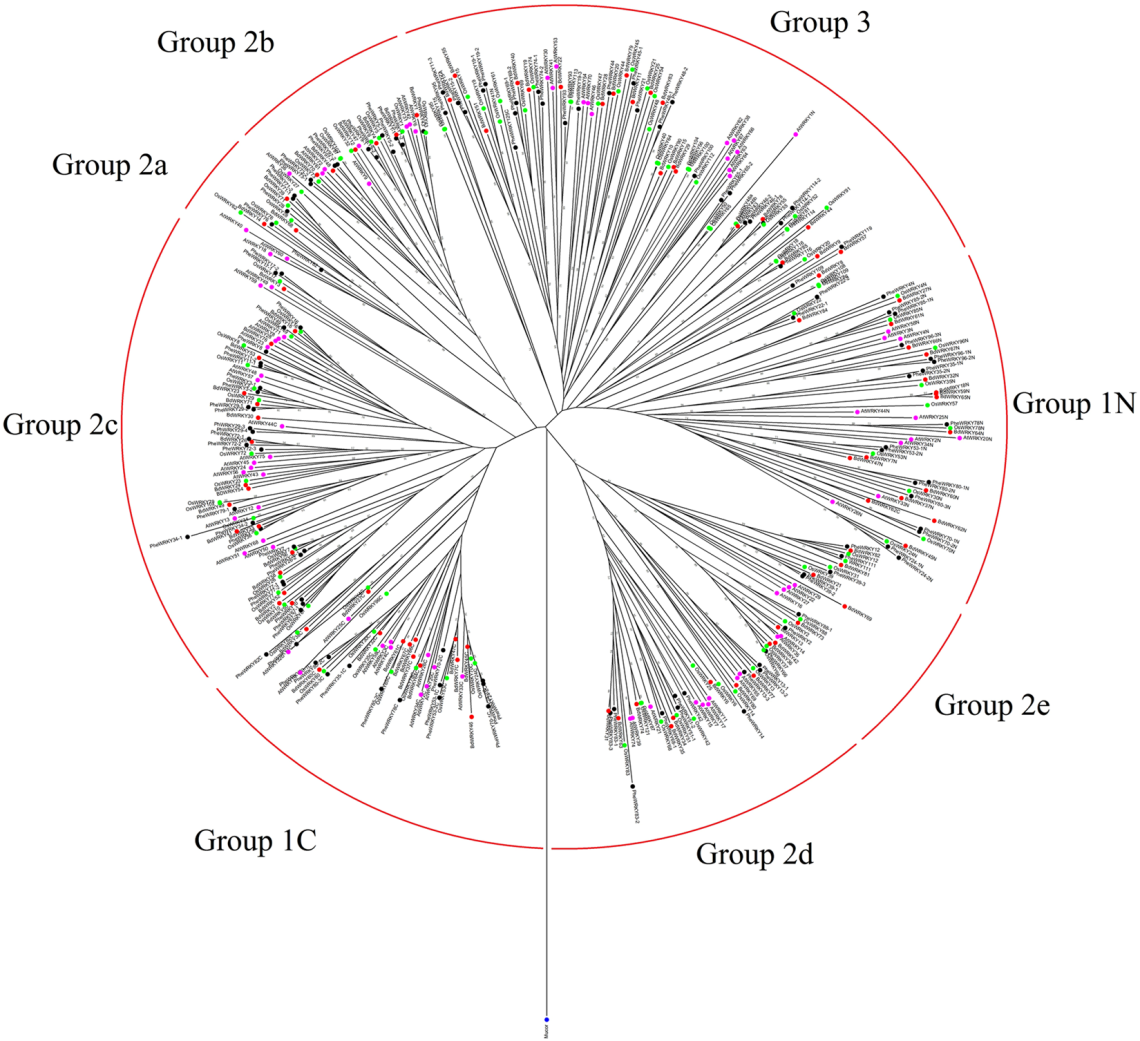
Rooted phylogenetic tree of the WRKY TF families. *A. thaliana*, *O. sativa*, *B. distachyon*, and *P. patens*. ‘Group 1 N’ and ‘Group 1 C’ indicate N-terminal or C-terminal WRKY domains, respectively, in group 1. *P. edulis*, *A. thaliana*, *O. sativa*, *B. distachyon*, and *P. patens* proteins are indicated by black, green, purple, red, and blue dots, respectively.

To examine the phylogenetic relationship among WRKY proteins in *P. edulis*, *A. thaliana*, *O. sativa*, and *Brachypodium distachyon*, we performed phylogenetic analyses of the WRKY domain sequences from all four species based on a neighbour-joining method using Mega 6 software. Because the N-terminal and C-terminal domains form distinct clusters, we designated the two domains as 1 N and 1 C, respectively (Fig. 3). The phylogenetic tree indicated a divergence between monocotyledons and dicotyledons. PheWRKY clearly shared more sequence similarity with OsWRKY and BdWRKY than with AtWRKY. We then used the bidirectional best hit (BBH) method, which is restricted to a 1:1 ratio of orthologues^31^, to arrange possible orthologues for PheWRKY from the three sequenced species mentioned above. Of the 121 sequences, we identified orthologues for 62 *PheWRKY* genes from at least one of the three plant species. The highest number of orthologues (52 members) was identified in *O. sativa*, whereas the lowest number (10 members) was detected in *A. thaliana* (Table S2).

These putative paralogous pairs accounted for 59.5%, 54.2%, 53.6% and 52.7% of the entire WRKY family in *P. edulis*, *O. sativa*, *B. distachyon* and *A. thaliana*, respectively. The median values of the divergence time for *P. edulis*, *O. sativa*, *B. distachyon* and *A. thaliana* were 13.9, 38.26, 42.84 and 23.25 mya, respectively. The divergence for most PheWRKY gene pairs (22 of 36) was approximately 6 to 15 mya, similar to the *P. edulis* whole-genome duplication event (7-12 mya)^4^ and much later than those of *O. sativa*, *B. distachyon* and *A. thaliana* (Table S5).

### Expression patterns of *PheWRKY* genes in different seedling tissues

We detected the expression of 81 *PheWRKY* genes in different tissues of three-month-old seedlings by RT-PCR. These selected genes covered all subgroups and included both up-regulated genes and down-regulated genes in flower development and shoot growth. Among them, *PheWRKY11-2*, *22-2*, *45-1*, *77-2*, *83-1* and *83-3* showed high expression levels in roots; *3-2*, *13-3* and *19-1* showed high expression levels in stems; and *26*, *67-1* and *71-2* were highly expressed in leaves (Figure S4).

### Expression analysis of the *PheWRKY* genes’ roles in flower development and shoot growth

**Figure 4:**
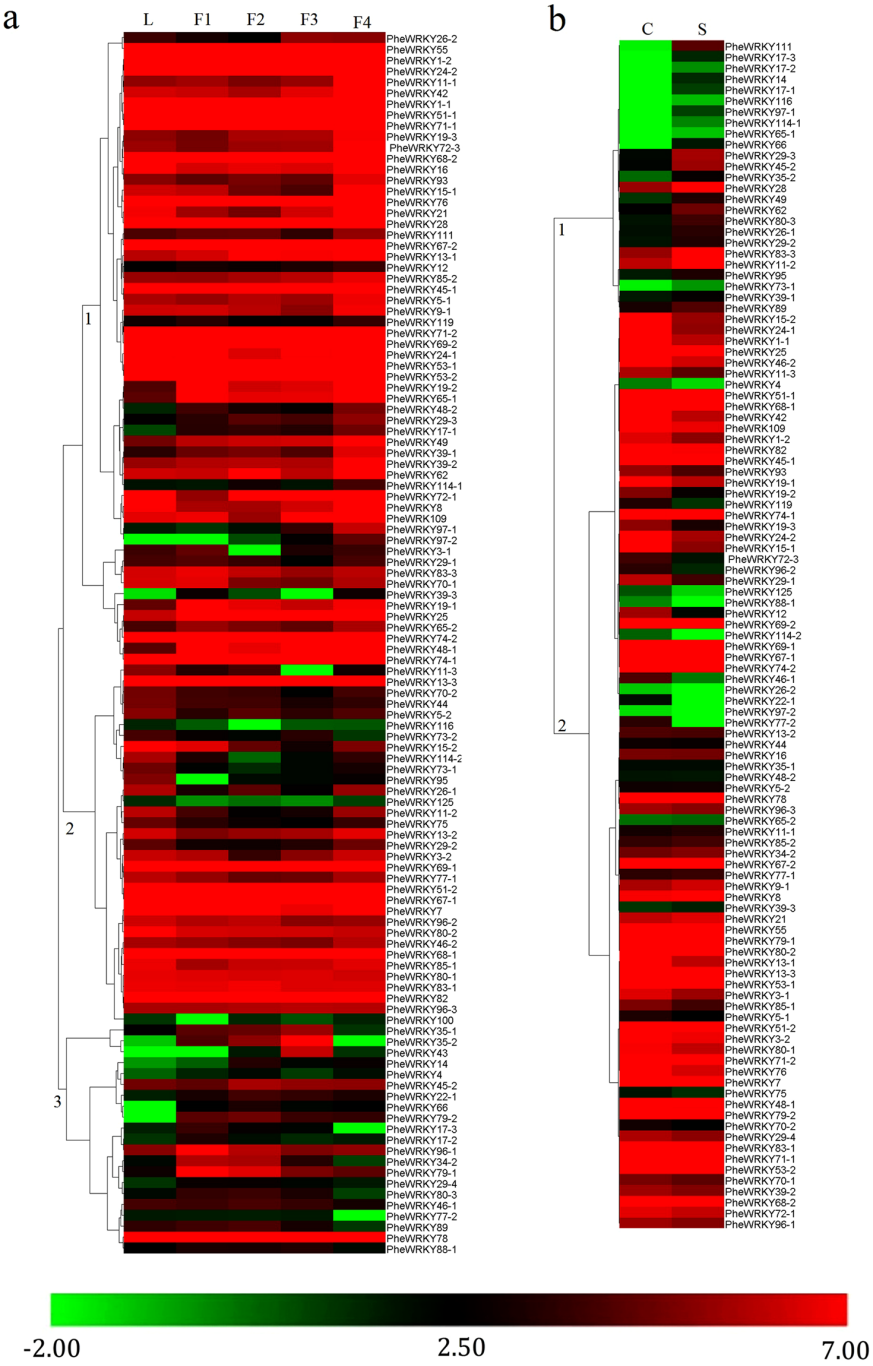
Expression profiles of the *PheWRKY* genes based on transcriptome data. The colour scale represents log2-transformed reads per kilobase per million (RPKM) values. Green indicates low expression, and red indicates high expression. **(a)** Dynamic expression profiles of four floral stages. F1, F2, F3, and F4 represent four different flowering developmental stages: the floral bud formation, inflorescence growing, blooming and embryo formation stages, respectively. **(b)** Expression profiles of the fast-growing shoot and culm.

We divided the *PheWRKY* genes into two groups (Fig. 4b) based on their expression in shoot and one-year-old culm. The expression levels of genes from cluster 1 were more abundant in the culm than in the shoot. In contrast, the expression of cluster 2 was significantly elevated in the shoot growth stage than in the one-year-old culm (CK). The genes *PheWRKY28, 29-3*, and *62* were significantly up-regulated during the shoot growth stage, suggesting that they play roles in the rapid elongation of the bamboo shoot (Table S6).

The expression profiles from the floral digital gene expression (DGE) data were divided into three cluster groups. Cluster 1 had genes with high expressions at stage F4. Cluster 2 genes exhibited steady expression with little fluctuation from stage F1 to F4. The genes from cluster 3 were initially up-regulated, reached their peak expression at stage F2 or F3, and declined thereafter. Compared with CK (leaf), *PheWRKY48-1* appeared to be preferentially expressed throughout the flowering process (Fig. 4a and Table S6). To validate the reliability of RNA-Seq and DGE data, quantitative reverse-transcription polymerase chain reaction (qRT-PCR) assays were randomly performed on four selected genes (Figure S5). As expected, in most cases, the expression trends of the selected genes corresponded to the presented data, indicating that the DGE and RNA-seq data were highly reliable (Figure S5).

A co-expression network was constructed to analyse the PheWRKY interactions in different biological processes. Of the top ten hub genes (degree ≥ 9) in the network, six genes, including *PheWRKY19-2*, *39-1*, *39-2*, *62*, *65-1*and *114-1*, were up-regulated in flower development (Figure S6).

### Expression profiles of PheWRKY genes under abiotic stress conditions

**Figure 5:**
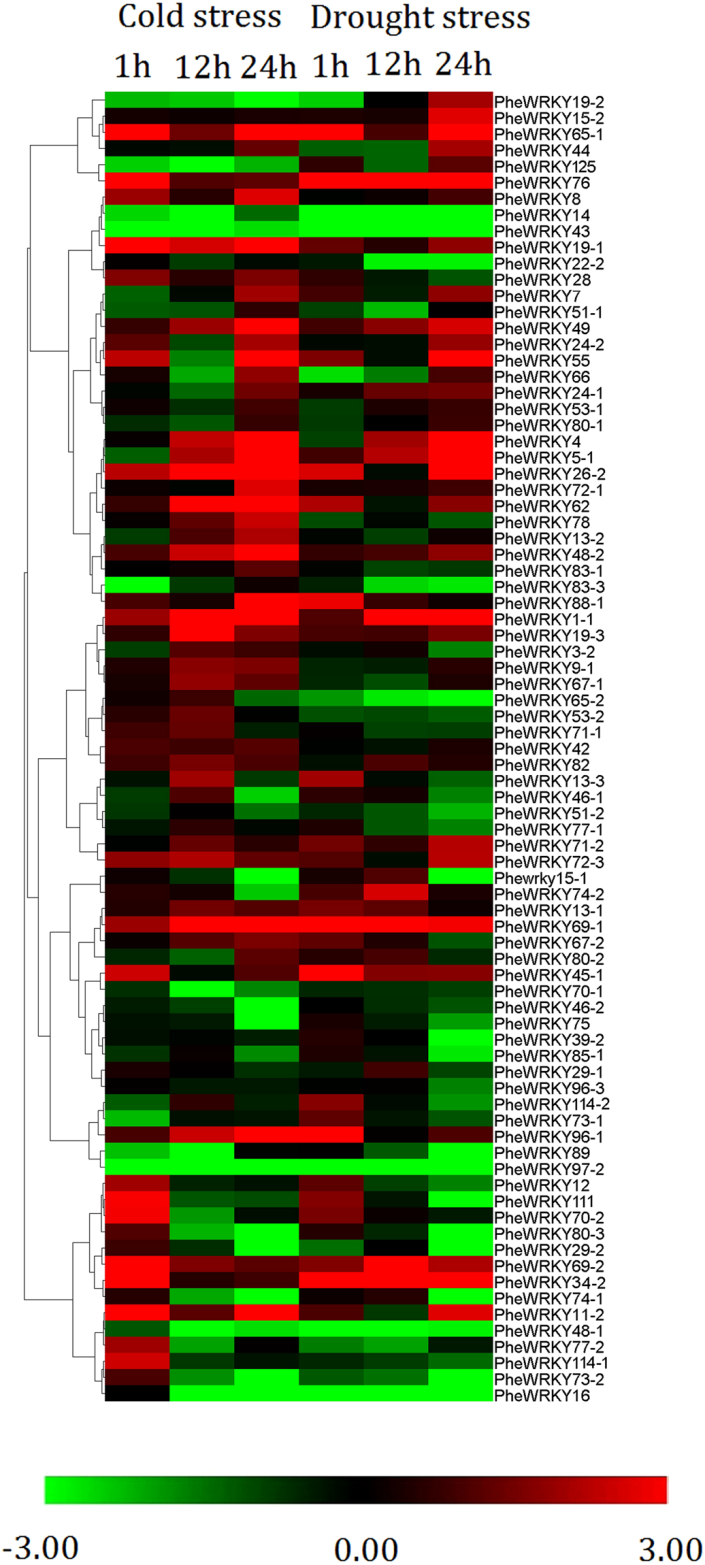
Expression profiles of WRKY genes under cold and drought stress. The expression results were obtained by qRT-PCR, and the relative expression levels of PheWRKY genes under various treatments compared to the controls were used for hierarchical cluster analysis with Cluster 3.0. qRT-PCR analysis was based on three biological replicates of each sample and three technical replicates of each biological replicate.

We detected the expression of 81 *PheWRKY* genes under drought and cold stress by qRT-PCR. Of these, 32 genes showed different degrees of up-regulation with at least one stress treatment, including 28 genes responding to cold treatment and 22 genes responding to drought treatment (Fig. 5). Interestingly, the most up-regulated genes in drought treatment (17 of the 22) were always up-regulated in response to cold treatment. The expression levels of *PheWRKY4*, *26-2*, *48-2*, *49*, *62*, *69–1*, *72–3* and *96–1* increased gradually and peaked at 12 h in response to cold treatment, whereas those of *PheWRKY11–2*, *111*, *34–2*, *45–1*, *114–1*, *69–2*, *70–2* and *76* rapidly accumulated at 1 h and then decreased to low levels. For drought treatment, *PheWRKY45–1*, *62*, *69–1*, *88–1*, *96–1*, *96–2* peaked at 1 h, and *PheWRKY4*, *5–1*, *26–2*, *15–2*, *19–2*, *44*, *49*, *55*, *65–1*, *71–2*, *72–3* peaked at 24 h. The expression profiles of *PheWRKY1*, *36*, *69–2* and *74–2* under drought treatment displayed parabolic trends and peaked at 12 h (Fig. 5 and Table S6).

Our expression analysis revealed that 54 genes were significantly induced by at least one abiotic stress (cold stress or drought stress) or physiological process (flower development or shoot growth). Of these WRKY genes, 7, 4, 17 and 3 were regulated by only cold stress, drought stress, flower development, and shoot growth, respectively (Figure S7).

### Overexpression of PheWRKY72–2 in Arabidopsis

**Figure 6:**
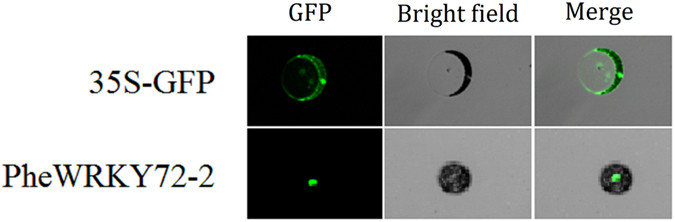
Subcellular localization analysis of PheWRKY72-2.

Subcellular location analysis indicated that green fluorescent protein (GFP)-tagged PheWRKY72–2 was located in the nucleus, in accordance with its function as a TF (Fig. 6).

**Figure 7:**
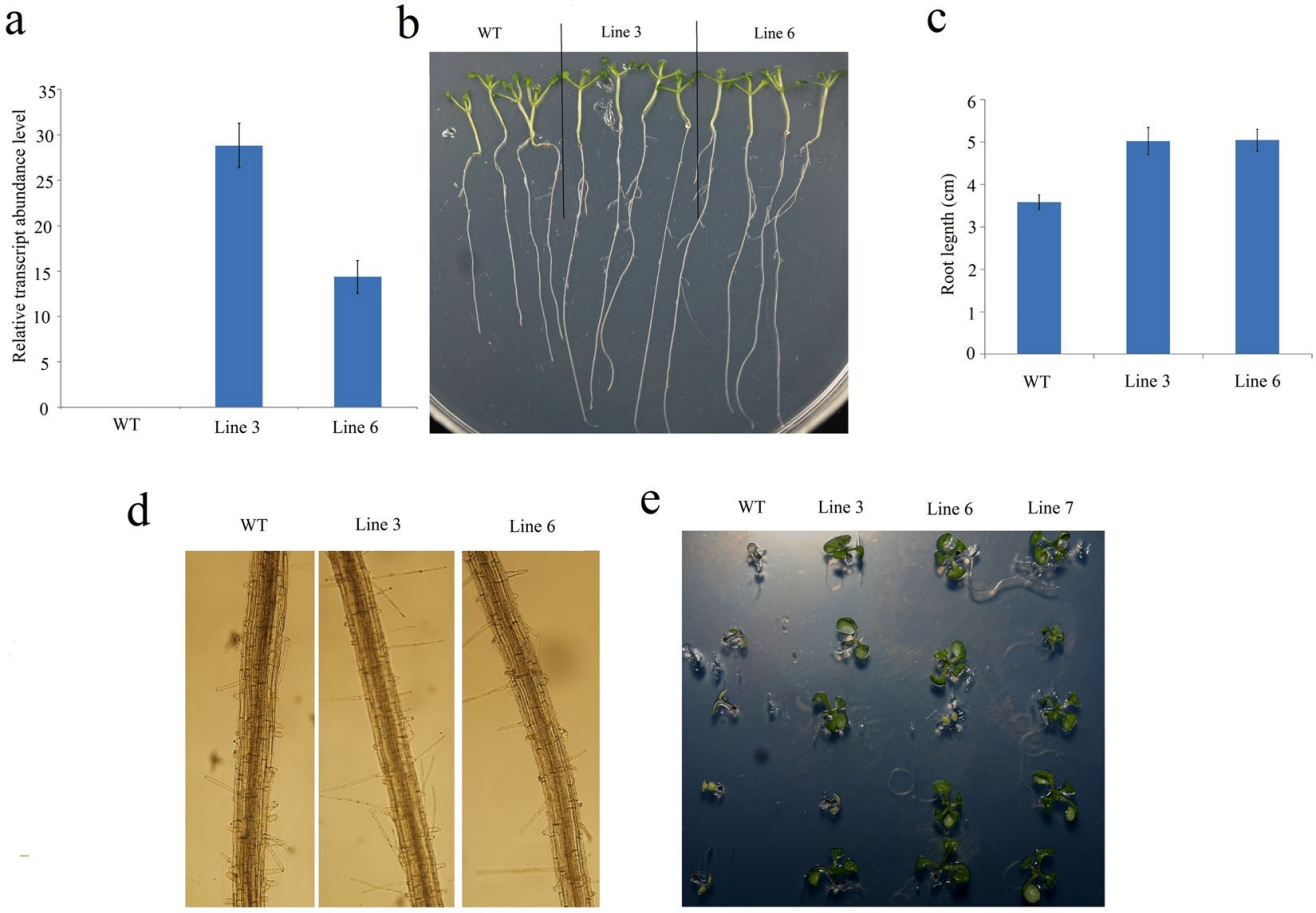
**(a)** Relative expression of *PheWRKY72* in transgenic lines. The expression of internal control gene (*β-tubuli)* was arbitrarily set at 1.0. **(b**) Response of 35S-PheWRKY72-2 *Arabidopsis* transgenic lines to drought stress (10% PEG). **(v)** The primary root lengths of WT and transgenic seedlings under drought stress (10% PEG). **(d)** Observation of root hairs under drought stress (10% PEG). **(e**) Response of the 35S-PheWRKY72-2 *Arabidopsis* transgenic lines to drought stress (20% PEG).

To further investigate the potential role of *PheWRKY72–2*, which was up-regulated under both drought and cold stress, *PheWRKY72–2* was transformed into *Arabidopsis* (wild type [WT]). Following qRT-PCR analysis of *PheWRKY72–2* in WT and transgenic lines confirmed that *PheWRKY72–2* performed high expression level in transgenic lines but no expression could be detected in WT (Fig. 7). Under cold stress treatment, no significant differences could be found between the WT and transgenic plants (data not shown). For simulated drought stress, transgenic *Arabidopsis* seeds were surface sterilized and germinated on Murashige and Skoog (MS) agar medium containing 10% and 20% polyethylene glycol (PEG) (Fig. 7b and e). The overexpression of *PheWRKY72–2* (T3 generation) resulted in significantly longer roots than those of WT plants on medium supplemented with 10% PEG (Fig. 7b and c). We investigated the root hairs further but found no visible difference between those of the WT and transgenic lines (Fig. 7d). However, when the PEG concentration reached 20%, the WT line began to exhibit etiolation symptoms, unlike the transgenic lines (Fig. 7e).

**Figure 8:**
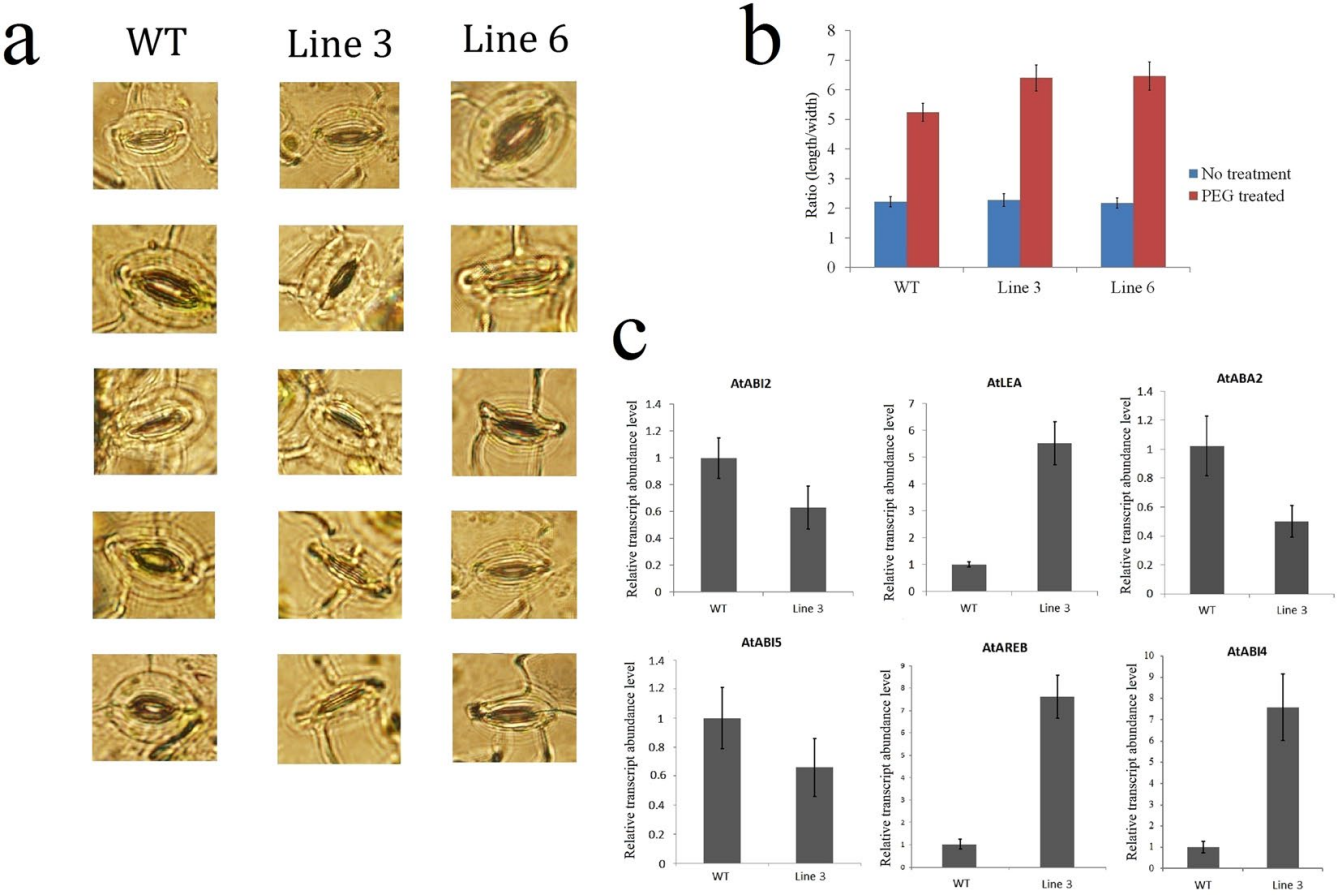
**(a)** Comparison of the stomatal aperture between transgenic and WT plants in response to drought stress. (**b**) Ratios of stomatal closure. The ratio of the length to the width was used as a measurement of stomatal closure. (**c**) Expression of ABA-signalling genes in WT and 35S-OsWRKY72 transgenic *Arabidopsis* under drought stress for 3 h. The expression of each gene in WT was arbitrarily set at 1.0. qRT-PCR analysis was based on three biological replicates of each sample and three technical replicates of each biological replicate.

We then compared the stomatal apertures under drought treatment because water loss mainly depends on stomatal regulation. To this end, we measured the stomatal apertures of WT and transgenic plants under control and drought treatments (3 h). The length and width of the stomata were determined, and the length/width ratio was used to reflect the degree of stomatal closure. Under normal growth conditions, no significant differences were detected (Fig. 8b). Under drought stress, the *PheWRKY72-2*ox lines showed a higher length/width ratio of stomata than the WT plants (Fig. 8a and b). These results suggest that stomatal closure in *PheWRKY72-2*ox plants is more sensitive to drought stress than that in WT plants. Under the drought treatment, OE-PheWRKY72-2 suppressed the expression of *AtABI2*, *AtABI5* and *AtABA2* and significantly increased the expression of *AtABI4*, *AtAREB* and *AtLEA* (Fig. 8c).

## Discussion

### Characterization of the *P. edulis* WRKY gene family

To facilitate the analysis of *P. edulis* WRKY gene evolution in group 1, we further classified *P. edulis* group 1 WRKY genes into two subgroups based on their zinc-finger motifs^32^. Except for PheWRKY61, whose zinc-finger motif of C_2_HC-C_2_HC was assigned to group 1b, all the other members of group 1 could be allocated to group 1a. PheWRKY61 probably evolved through an intra-molecular duplication event of a group 3 WRKY domain that had already evolved to have the C_2_HC type zinc-finger. PheWRKY35, 85-1, 96-1, 96-2 and 96-3 all contained two WRKY domains but lacked a complete zinc-finger in their C-terminal domains. The closing genetic relationship of the N-terminal WRKY domains among PheWRKY85-1, 96-1, 96-2 and 96-3 determined based on the phylogenetic tree suggests that these sequences underwent some similar evolutionary events. Domain acquisition and domain loss events appear to have shaped the WRKY family of proteins^33^. Thus, these five genes might have arisen when a two-domain WRKY gene lost the zinc-finger motif on its C-terminal domain during evolution. Small variations in the WRKYGQK sequence are described for some OsWRKY proteins (accounting for 11 OsWRKY domains)^32^. These variations are also very common (11 domains) in *B. distachyon*, but only three *A. thaliana* members had variant heptapeptide sequences (WRKYGKK) (Table S4). In moso bamboo, we found that amino acid substitutions in the conserved heptapeptide signature (15 domains) were much more common than in the three model plants.

In previous reports, bamboo was considered to have a tetraploid origin. Approximately 7-12 mya, *P. edulis* experienced a long progression from tetraploidy to diploidy^4^. *P. edulis* carries two duplicates as that of *O. sativa* gene model sets^4^. For *PheWRKY* genes, a similar phenomenon was always observed. Although *P. edulis* suffered a large-scale gene loss event after the whole-genome duplication, 36 putative paralogous pairs of *PheWRKY* genes remained, far exceeding the numbers in *A. thaliana, O. sativa* and *B. distachyon*. Furthermore, most duplication events of WRKY paralogous pairs in this species occurred 6-15 mya, much later than those of the three model plants mentioned above. All of our results suggest that the recent whole-genome duplication, which was likely linked to polyploidy events, played an important role in the WRKY family expansion.

### Diverse PheWRKYs are involved in various biological processes

WRKY TFs regulate a range of biological processes and plant responses to various abiotic stressors. At least 54 *OsWRKY* genes in *O. sativa* ^34^ and 23 *CsWRKY* genes in *Cucumis sativus* are differentially expressed under abiotic stresses^35^. Similarly, 55 *VvWRKY* genes differentially respond to at least one abiotic stress treatment^36^. We profiled the expression of 80 *PheWRKY* genes in plants subjected to cold and drought stress and detected 31 *P. edulis WRKY* genes with significantly higher expression in response to at least one abiotic stress using real-time PCR, indicating that, in *P. edulis*, the *PheWRKY* genes function in the abiotic stress response. The overexpression of *PheWRKY72-2* enhanced plant tolerance to drought stress. In *Arabidopsis*, *AtWRKY54* and *AtWRKY70* enhance plant tolerance to osmotic stress by regulating the size of the stomatal aperture^37^. Various environmental stresses result in the rapid accumulation of ABA, leading to stomatal closure, which reduces water loss by transpiration^38^. Recent reports showed that *AtWRKY46* participated in the feedforward inhibition of lateral root inhibition via regulation of ABA signaling and auxin homeostasis under osmotic/salt stress treatment^39, 40^. Besides, WRKY46 is regulated by light and modulates starch metabolism and ROS levels to inhibit stomatal closing under osmotic stress treatment by another independent way. Unlike the overexpression line of *AtWRKY46* whose stomatal closing is impaired under osmotic stress treatment^40^, the transgenic lines of *PheWRKY72-2* showed enhanced stomatal closure under drought stress by comparing with the WT line. ABA controls stomatal movement via a dual mechanism. Regulation can occur via the biochemical effects of ABA on guard cells^38^. Salt stress is often accompanied by drought stress, and both cause water deprivation through ABA-dependent and ABA-independent pathways^41^. Our results showed that *OE-PheWRKY72-2* significantly promoted the expression of *AtLEA*, *AtAREB* and *AtABI4* and repressed the expression of *AtABI2*, *AtABI5* and *AtABA2*. Plants with up-regulated ABI1/2 expression exhibited decreased stress tolerance, whereas plants with down-regulated ABI1/2 expression exhibited increased stress tolerance^42^. Reactive oxygen species (ROS), such as superoxide, hydrogen peroxide and hydroxyl radicals, play a dual role in plants: They act as necessary signalling molecules but can cause damage to plant cells when overproduced under stress conditions^43^. The constitutive expression of *AtAREB1* in *Arabidopsis* modulates ROS accumulation and endogenous ABA levels to improve drought tolerance^43^. In plants, the presence of late embryogenesis abundant (LEA) proteins has been associated with cellular tolerance to dehydration, which may be induced by freezing, saline conditions, or drying^44^. Thus, up-regulating *AtLEA* might improve stress tolerance in transgenic plants.

Compared with their expression in nonflowering moso bamboo leaves (CK), the significant up-regulation of *PheWRKY19-1*, *19-2*, *35-2*, *48-1* and *65-1* during flower development indicated their potential roles in morphogenesis and organ development. WRKY TFs associated with senescence^45–47^ and stress responses^48^ were significantly up-regulated in the panicles of *P. edulis*. Under normal circumstances, *P. edulis* rarely flowers, and the plants often flower concurrently and die collectively after flowering. However, collective death after flowering in a large area is usually associated with external environmental changes, such as climatic variation or the over-exploitation of bamboo^1^. The overexpression of *AtWRKY75* or *OsWRKY72* causes early flowering in *Arabidopsis* ^49^, and the overexpression of *GsWRKY20* accelerates plant flowering by regulating flowering-related genes (i.e., flowering locus T [FT] and CONSTANS [CO]) and floral meristem identity genes (i.e., activator protein 1 [AP1], SEPALLATA 3 [SEP3] and AGAMOUS [AG])^48^. The involvement of WRKY factors in floral induction may be induced by signalling hormones, such as salicylic acid^7, 50^, jasmonic acid^45, 46^ or gibberellic acid^10^. We found that *PheWRKY 28*, *29-3*, *49*, *62*, *80-3* and *111* have significant expression levels in growing shoots but not in culms. *OsWRKY78* has positive effects on stem elongation and cell length, as determined by transgenic studies^51^. The up-regulation of several WRKY genes in shoot growth and development indicate that many WRKY genes function in the rapid growth and elongation of *P. edulis* shoots. In summary, *PheWRKY* genes play varied roles in different biological processes in *P. edulis*.

### The functional conservative and divergence of orthologous genes

We further analysed the correlations of orthologous pairs under various processes. In *Arabidopsis*, the expression pattern of *AtWRKY53* was identified as a leaf senescence signal^44^. Constitutively overexpressing *AtWRKY53* under the control of the CaMV35S promoter accelerated the senescence symptoms, whereas *WRKY53* RNAi and knock-out lines exhibited delayed development and senescence^45^. The orthologues, *PheWRKY15-1* (4E-039) and *PheWRKY74-1* (2E-31), were always found in high abundance in leaves collected from flowering plants (flowering implies senescence and death in *P. edulis*) compared to their abundance in leaves collected from other growth stages (Figure S8), indicating that there are conserved roles between WRKY homologues in leaf senescence. Moreover, we compared the expression trends of *PheWRKY* genes with those of their possible orthologues from *A. thaliana*, *O. sativa* ^34^, and *B. distachyon* ^46^ under cold and drought stress. Although the sample times and experimental conditions were different, our data revealed that some *P. edulis* homologues in other model plants showed an entirely opposite expression trend. *PheWRKY85-1* shares 87% sequence similarity with *OsWRKY85*; however, *OsWRKY85* is up-regulated under drought stress, whereas *PheWRKY85-1* is down-regulated. The function of these segregated duplicated genes (*PheWRKY85-1* and *OsWRKY85*) may have been altered during evolution^34^.

## Conclusions

In this study, a total of 121 WRKY genes were identified in *P. edulis*. The expression profiles derived from DGE, RNA-seq and qRT-PCR analyses indicated that *PheWRKY* involved in various abiotic stress. The overexpression of *PheWRKY72-2* in *Arabidopsis* increased drought tolerance by functioning as a positive regulator of stomatal closure. Better understanding this gene family and its potential involvement in growth, development and stress responses will facilitate further research on the evolutionary history and biological functions of the PheWRKY gene family.

## Materials and Methods

### Plant materials, growth conditions, and treatments

Samples of *P. edulis* culms were harvested in Huoshan County (116° 10′; 31° 12′), Anhui province from spring to autumn of 2014. Flowering plant samples from different developmental stages of the flowering process (F1-floral bud formation, F2-inflorescence development, F3-anthesis, and F4-embryo formation) were collected in Guilin city (110° 17′; 25° 048′), Guangxi Zhuang Autonomous Region, from April to August in 2014. Plants were sampled from sites suitable for bamboo growth that were free of insect pests and artificial destruction. Samples were collected according to previously described methods^1, 3^.

For the abiotic stress treatment, *P. edulis* seeds were germinated in culture dishes lined with soggy filter paper for 1 week and then transferred into four seed pots containing approximately 0.5 kg of vermiculite. The seedlings were grown in an artificial climate chamber with long-day conditions (16-hrs light, 8-hrs dark) at 26 °C in the light and 18 °C in the dark. Drought stress was created by irrigating the plants with media containing 18% (v/v) PEG. Cold stress was created by placing plants in a 4 °C lighted growth chamber; the other conditions were the same as described above. The leaves from three seedlings were harvested at 0, 0.5, 1, 3, 6, 12 and 24 hrs after abiotic stress treatment, rapidly frozen in liquid nitrogen and stored at −80 °C prior to use.

### RNA isolation, reverse transcription and gene expression analysis

Total RNA was extracted using Trizol (Invitrogen, USA). First-strand cDNA synthesis was conducted with approximately 1 μg of RNA using the reverse transcriptase AMV (Promega, Madison, Wisconsin, USA). qRT-PCR was performed using the fluorescent intercalating dye Light Cycler 480 SYBR Green I Master Mix (Roche, Mannheim, Germany) on a Light Cycler 480 (Roche, Rotreuz, Switzerland). Primers were designed using Primer 3 software and Oligo dT7 (Table S7). According to the manufacturer’s instructions, the 20-μL reaction mixture contained 0.4 μL (10 μM) of each primer, 1.5 μL (30 ng) of cDNA and 10 μL of SYBR Green I Master Mix. *TIP41* (tonoplast intrinsic protein 41)^52^ and *β*-tubulin were selected as internal controls. All reactions—technical and biological—were performed in triplicate. The ΔCT and ΔΔCT values were calculated by the formulas ΔCT = CT target − CT reference and ΔΔCT = ΔCT treated sample −ΔCT untreated sample, respectively.

### Gene sequence verification

A full-length fragment of *PheWRKY9-1* was cloned from *P. edulis* cDNA by RT-PCR using the following primer pair: 5′-ATGGAGGCGGTATCGGCGGT-3′ and 5′-TTAGATCGATCTGCGAGGTGCGGTC-3′.

### Database searches

The annotated WRKY genes of *A. thaliana*, *O. sativa*, and *B. distachyon* were obtained from The *Arabidopsis* Information Resource (TAIR; http://www.arabidopsis.org/), the rice genome annotation project (http://rice.plantbiology.msu.edu/index.html) and the *B. distachyon* WRKY database (http://www.igece.org/WRKY/BrachyWRKY/BrachyWRKYIndex.html). All annotated WRKY genes of *P. edulis* were downloaded from the National Center for Gene Research (http://202.127.18.221/bamboo/down.php). To avoid missing potential members of the WRKY family, we created a local blast database using the CDS sequence that we obtained from the *P. edulis* CDS database and novel transcripts that we obtained from RNA-seq data. The novel transcripts were assembled in a GTF file (exported by Cufflink based on a series of transcriptome sequencing results), and the *P. edulis* genome data were analysed using Perl. Published *AtWRKY* sequences and *OsWRKY* were retrieved and used as queries in blastn searches against the CDS database, and sequences were selected as candidate genes if their E-value was ≤ −10. The Pfam (http://pfam.sanger.ac.uk/search) and Smart (http://smart.emblheidelberg.de/) databases were used to confirm each predicted WRKY sequence. The pI and molecular weight were estimated using the Compute pI/Mw tool from ExPASy (http://web.expasy.org/compute pi).

### Multiple sequence alignment and phylogenetic tree construction

Multiple alignments of the amino acid sequences were performed using the ClustalX 2.1 program with default settings^53^. A phylogenetic tree based on sequence alignment was generated using MEGA 6.0 (http://www.megasoftware.net/) by the neighbour-joining method^54^. In addition, the BBH method^31^ was used to arrange possible orthologues and paralogues.

### Gene structure and conserved motif analysis

The gene structure based on full-length CDS alignments with relevant genomic sequences was investigated using the online service of the Gene Structure Display Server^55^. MEME^56^ was used to identify motifs in the *PheWRKY* sequences.

### Estimation of the duplication time in paralogous pairs

The K_a_ and K_s_ values of the paralogous genes were computed by the DNASP program. The synonymous substitution rate (K_s_) was considered as a proxy for time to estimate the dates of the segmental duplication events. The formula T = K_s_/2λ was used to o calculate the approximate date of the duplication event and the λ in formula represented for clock-like rates of synonymous substitution. The estimated clock-like rate for *P. edulis*, *O. sativa* and *B. distachyon* were 6.5 × 10^−9^ substitutions/synonymous site/year and that that for *A. thaliana* was 1.5 × 10^−8^ substitutions/synonymous site/year^57, 58^.

### DGE data, transcriptome data analysis, and co-expression network generation

The DGE data of four different developmental stages of flowering (floral bud formation, inflorescence growing, blooming and embryo formation) and the transcriptomic data of fast-growing shoots at two developmental stages (shoots mixed by six unearthed heights [10, 50, 100, 300, 600, and 900 cm] and mature culm) were sequenced in our previous study^1, 3^ and used for *PheWRKY* gene expression analyses. For floral development, the DGE data of nonflowering, moso bamboo leaves (CK) were regarded as the reference gene database^1^. For shoot growth, the transcriptome data of one-year-old culms constituted the reference gene database^3^. The expression levels of *PheWRKY* genes at different growth stages were hierarchically clustered based on Euclidean distance with complete linkage found in Cluster 3.0. A gene was considered up-regulated if it had a false discovery rate (FDR) ≤0.05 and│log_2_ fold change│ ≥ 2.

The microarray data and RNA-seq data were used for coexpression analysis via the WGCNA package. The co-expression correlation was then calculated using Pearson’s correlation with R^2^ ≥ 0.95. The network picture was created using Cytoscape.

### Overexpression of *PheWRKY72-2*

The complete open reading frame (ORF) of the *PheWRKY72-2* (FP101056.1) gene cloned by Chen^59^ in our previous work was inserted into the pGEM-T Easy plasmid vector using the following primers: forward, 5′-TGCTCTAGAATGGAAGCCTACCCTATGCT-3′; reverse, 5′-CCGGAATTCTCAGTGGAACCGGCCAGAC-3′. TIANGEN DNA Polymerase (TIANGEN biological company, China) was used to amplify the *PheWRKY72-2* gene. The PCR parameters were 94 °C for 5 min, followed by 28 cycles of 94 °C for 30 s, 62 °C for 1 min and 72 °C for 1 min. The PCR products was digested with *BamH* I and *Xba* I and then cloned into pGEM-T Easy (Promega, USA) after gel extraction. The coding region of the *PheWRKY72-2* cDNA was cloned into the pCAMBIA 2300 vector under the control of the CaMV 35 S promoter and CAMV terminator. The constructed plasmid was named pCAMBIA2300-PheWRKY72-2 and confirmed by the chain-termination method on an ABI 3100 automated sequencer (USA).

The construct vector was introduced into WT *Arabidopsis* plants (Columbia-0) by *Agrobacterium*-mediated transformation. Transgenic plants were selected on kanamycin, and the first generation of transgenic plants was examined in terms of their phenotypes. At least 10 independent transgenic plants exhibiting severe phenotypes were selected, subjected to phenotypic characterization and screened for the transgene. Transgenic *Arabidopsis* seeds (T3 generation) and WT seeds were cultured with 10% and 20% PEG solution on MS agar medium. One-week-old seedlings were used to observe the phenotype. Three-week-old transgenic plants and WT which growth in normal growth condition were treated with PEG solution for 3 h and further used for RT-PCR experiment and stomatal closure observation.

### Subcellular localization

The subcellular localization of PheWRKY72-2 was performed by transfecting GFP-tagged PheWRKY72-2 into *Arabidopsis* sheath protoplasts. The full-length cDNA of *PheWRKY72-2* was fused in frame with the GFP cDNA and ligated between the CaMV 35 S promoter and the nopaline synthase terminator. The fluorescence signals in transfected protoplasts were examined by a confocal laser scanning microscope (Leica Microsystems).

### Availability of supporting data

All sequencing data were deposited in the Short Read Archive at NCBI under accession number SRR961047, SRR1187864 and SRR1185317.

## Electronic supplementary material

Supplementary data
