# Supplementary material for: Characterization and expression analysis of the WRKY gene family in moso bamboo
Source: Sci Rep. 2017 Jul 27;7:6675. doi: 10.1038/s41598-017-06701-2 (PMC5532226; doi:10.1038/s41598-017-06701-2)
Supplement: Supplementary file 1 — Supplementary data [file 41598_2017_6701_MOESM1_ESM.pdf]

Supplementary data

Characterization and expression analysis of the *WRKY* gene family in moso bamboo

Long Li[1], Shaohua Mu[1], Zhanchao Cheng[1], Yuanwen Cheng[1], Ying Zhang[1], Ying Miao[2], Chenglin Hou[3], Xueping Li*[1], Jian Gao*[1]

1. International Center for Bamboo and Rattan, Key Laboratory of Bamboo and Rattan Science and Technology, State Forestry Administration, Beijing, 100102, People's Republic of China
2. Center for Molecular Cell and Systems Biology, College of Life Sciences, Fujian Agriculture and Forestry University, Fuzhou 350002, People's Republic of China
3. Department of Microbiology, College of Life Science, Capital Normal University, Beijing 100048, People's Republic of China

* Corresponding author Xueping Li Email: lxp@icbr.ac.cn Fax: 0086-10-84789805 Telephone: 0086-010-84789805. Jian Gao Email: gaojian@icbr.ac.cn Fax: 0086-10-84789900 Telephone: 0086-010-84789801.

Supplementary Table S1. Gene models of novel identified PheWRKY genes.

Gene	Scaffold	Software	Exon	Begin site	End site	Strand	Comment
PheWRKY2	PH01002667	Cufflinks	exon	124171	124983	+	gene_id "XLOC_041653"; transcript_id "TCONS_00100293"; exon_number "1"; oId "CUFF.37351.1"; class_code "u";
PheWRKY2	PH01002667	Cufflinks	exon	125841	126292	+	gene_id "XLOC_041653"; transcript_id "TCONS_00100293"; exon_number "2"; oId "CUFF.37351.1"; class_code "u";
PheWRKY10	PH01069729	Cufflinks	exon	11	309	-	gene_id "XLOC_054786"; transcript_id "TCONS_00125853"; exon_number "1"; oId "CUFF.49235.1"; class_code "u";
PheWRKY10	PH01069729	Cufflinks	exon	404	688	-	gene_id "XLOC_054786"; transcript_id "TCONS_00125853"; exon_number "2"; oId "CUFF.49235.1"; class_code "u";
PheWRKY34-1	PH01000922	Cufflinks	exon	401638	402071	-	gene_id "XLOC_025331"; transcript_id "TCONS_00062338"; exon_number "1"; oId "CUFF.22627.1"; class_code "u";
PheWRKY34-1	PH01000922	Cufflinks	exon	402199	402260	-	gene_id "XLOC_025331"; transcript_id "TCONS_00062338"; exon_number "2"; oId "CUFF.22627.1"; class_code "u";
PheWRKY34-1	PH01000922	Cufflinks	exon	402364	402855	-	gene_id "XLOC_025331"; transcript_id "TCONS_00062338"; exon_number "3"; oId "CUFF.22627.1"; class_code "u";
PheWRKY36	PH01000023	Cufflinks	exon	1155443	1155859	+	gene_id "XLOC_002126"; transcript_id "TCONS_00005299"; exon_number "1"; oId "CUFF.1882.1"; class_code "u";
PheWRKY36	PH01000023	Cufflinks	exon	1155936	1156012	+	gene_id "XLOC_002126"; transcript_id "TCONS_00005299"; exon_number "2"; oId "CUFF.1882.1"; class_code "u";
PheWRKY36	PH01000023	Cufflinks	exon	1156174	1156338	+	gene_id "XLOC_002126"; transcript_id "TCONS_00005299"; exon_number "3"; oId "CUFF.1882.1"; class_code "u";
PheWRKY36	PH01000023	Cufflinks	exon	1166061	1166487	+	gene_id "XLOC_002126"; transcript_id "TCONS_00005299"; exon_number "4"; oId "CUFF.1882.1"; class_code "u";
PheWRKY72-2	PH01003536	Cufflinks	exon	64316	64898	+	gene_id "XLOC_045802"; transcript_id "TCONS_00109438"; exon_number "1"; oId "CUFF.41115.1"; class_code "u";
PheWRKY72-2	PH01003536	Cufflinks	exon	66606	67022	+	gene_id "XLOC_045802"; transcript_id "TCONS_00109438"; exon_number "2"; oId "CUFF.41115.1"; class_code "u";

Supplementary Table S2. putative orthologous pairs of four plant (moso bamboo, *A. thaliana*, *O. sativa* and *B. distachyon*) and their expression trend under abiotic stresses.

Name	Cold stress	Drought stress	Score (bits)	E-value	orthology	*A. thaliana*	Cold stress	Drought stress	*O. sativa*	Cold stress	Drought stress	*B. distachyon*	Cold stress	Drought stress
PheWRKY1-1	U	U	853	0.00E+00	OsWRKY1	AtWRKY6		D	OsWRKY1			BdWRKY5		D
PheWRKY1-2			517	1.00E-149	OsWRKY1									
PheWRKY2			239	6.00E-66	OsWRKY2									
PheWRKY3-1	U	U	348	8.00E-99	OsWRKY3				OsWRKY3					
PheWRKY3-2			331	1.00E-93	OsWRKY3							BdWRKY23		D
PheWRKY4	U	U	504	1.00E-145	OsWRKY4				OsWRKY4			BdWRKY27		D
PheWRKY5-1	U	U	422	1.00E-121	OsWRKY5				OsWRKY5		U			
PheWRKY5-2			237	3.00E-65	OsWRKY5									
PheWRKY7	U		310	2.00E-87	OsWRKY7				OsWRKY7		U	BdWRKY56		
PheWRKY8	U		327	2.00E-92	OsWRKY8				OsWRKY8					
PheWRKY9-1			380	1.00E-108	OsWRKY9				OsWRKY9		U	BdWRKY4		
PheWRKY9-2			263	4.00E-73	OsWRKY9									
PheWRKY10			168	6.00E-45	OsWRKY10									
PheWRKY11-1	U		535	1.00E-155	OsWRKY11				OsWRKY11			BdWRKY53		
PheWRKY11-2	U	U	511	1.00E-148	OsWRKY11									
PheWRKY11-3			78	9.00E-18	OsWRKY11									
PheWRKY12			411	1.00E-118	OsWRKY12				OsWRKY12		U	BdWRKY82		D
PheWRKY13-1			449	1.00E-129	OsWRKY13				OsWRKY13			BdWRKY77		
PheWRKY13-2	U		444	1.00E-128	OsWRKY13	AtWRKY65	U	U						
PheWRKY13-3			258	1.00E-71	OsWRKY13									
PheWRKY14	D	D	236	5.00E-65	OsWRKY14				OsWRKY14			BdWRKY80	U	
PheWRKY15-1	D	D	318	1.00E-89	OsWRKY15									
PheWRKY15-2		U	236	3.00E-65	OsWRKY15							BdWRKY55	U	
PheWRKY16			421	1.00E-120	OsWRKY16				OsWRKY16			BdWRKY16	U	
PheWRKY17-1			273	9.00E-76	OsWRKY17									
PheWRKY17-2			258	2.00E-71	OsWRKY17									
PheWRKY17-3			65	8.00E-13	OsWRKY17									
PheWRKY19-1	U		270	2.00E-75	OsWRKY19									
PheWRKY19-2	D		267	2.00E-74	OsWRKY19									
PheWRKY19-3			109	9.00E-27	OsWRKY19							BdWRKY11		
PheWRKY21			273	4.00E-76	OsWRKY21				OsWRKY21			BdWRKY25		
PheWRKY22-1			327	2.00E-92	OsWRKY22				OsWRKY22			BdWRKY84		D
PheWRKY22-2		D	298	1.00E-83	OsWRKY22									
PheWRKY24-1			902	0.00E+00	OsWRKY24				OsWRKY24			BdWRKY45		
PheWRKY24-2			890	0.00E+00	OsWRKY24									
PheWRKY25			76	3.00E-17	OsWRKY25									
PheWRKY26-1	U	U	253	2.00E-70	OsWRKY7							BdWRKY58		D
PheWRKY26-2	U	U	205	1.00E-67	OsWRKY7									
PheWRKY28			377	1.00E-107	OsWRKY71				OsWRKY71					
PheWRKY29-1			346	3.00E-98	OsWRKY29									
PheWRKY29-2	D	D	330	3.00E-93	OsWRKY29				OsWRKY29			BdWRKY71		
PheWRKY29-3			152	7.00E-40	OsWRKY29									
PheWRKY29-4			145	8.00E-38	OsWRKY29									
PheWRKY34-1			284	1.00E-79	OsWRKY34									

PheWRKY34-2	U	U	272	5.00E-76	OsWRKY36	AtWRKY12			OsWRKY36			BdWRKY41	D
PheWRKY35-1			725	0.00E+00	OsWRKY30								
PheWRKY35-2			557	1.00E-161	OsWRKY35								
PheWRKY36			348	6.00E-99	OsWRKY36							BdWRKY33	
PheWRKY39-1			418	1.00E-120	OsWRKY39				OsWRKY39	D		BdWRKY21	
PheWRKY39-2		D	332	4.00E-94	OsWRKY39								
PheWRKY39-3			229	6.00E-63	OsWRKY39								
PheWRKY42			272	5.00E-76	OsWRKY42				OsWRKY42				
PheWRKY43	D	D	664	0.00E+00	OsWRKY43				OsWRKY43			BdWRKY1	D
PheWRKY44		U	272	8.00E-76	OsWRKY44				OsWRKY44	D		BdWRKY20	
PheWRKY45-1	U	U	318	1.00E-89	OsWRKY45				OsWRKY45	D			
PheWRKY45-2			62	9.00E-13	OsWRKY45								
PheWRKY46-1	D		363	1.00E-103	OsWRKY46a								
PheWRKY46-2	D		308	1.00E-86	OsWRKY46a							BdWRKY43	
PheWRKY48-1	D	D	301	1.00E-84	OsWRKY48							BdWRKY83	D
PheWRKY48-2	U		276	4.00E-77	OsWRKY48								
PheWRKY49	U	U	405	1.00E-116	OsWRKY49				OsWRKY49		U	BDWRKY76	
PheWRKY51-2		D	523	1.00E-151	OsWRKY51				OsWRKY51	D			
PheWRKY51-1		D	177	2.00E-47	OsWRKY51	AtWRKY11						BdWRKY34	D
PheWRKY65-1	U	U	207	3.00E-56	OsWRKY50				OsWRKY50	D	U		
PheWRKY65-2		D	177	2.00E-47	OsWRKY50								
PheWRKY53-1			709	0.00E+00	OsWRKY53	AtWRKY33	U	D	OsWRKY53		U	BdWRKY47	
PheWRKY53-2			486	1.00E-140	OsWRKY53								
PheWRKY55	U	U	410	1.00E-117	OsWRKY55				OsWRKY55	D		BdWRKY78	
PheWRKY62	U		213	3.00E-58	OsWRKY62				OsWRKY62	D			
PheWRKY66	D	D	578	1.00E-167	OsWRKY66	AtWRKY14			OsWRKY66	D			
PheWRKY67-1			241	1.00E-66	OsWRKY67				OsWRKY67	D		BdWRKY50	D
PheWRKY67-2			239	4.00E-66	OsWRKY67								
PheWRKY68-1			506	1.00E-146	OsWRKY68				OsWRKY68	D		BdWRKY35	
PheWRKY68-2			184	4.00E-50	OsWRKY68								
PheWRKY69-1	U	U	342	5.00E-97	OsWRKY69				OsWRKY69		U	BdWRKY40	D
PheWRKY69-2	U	U	328	9.00E-93	OsWRKY69								
PheWRKY70-1	D		658	0.00E+00	OsWRKY70				OsWRKY70		U	BdWRKY45	
PheWRKY70-2	U		627	0.00E+00	OsWRKY70								
PheWRKY71-1			478	1.00E-137	OsWRKY71	AtWRKY40	U	U				BfWRKY39	
PheWRKY71-2		U	468	1.00E-134	OsWRKY71								
PheWRKY72-1	U		290	3.00E-81	OsWRKY72	AtWRKY75			OsWRKY72			BdWRKY26	
PheWRKY72-2			209	4.00E-57	OsWRKY72								
PheWRKY72-3	U	U	195	6.00E-81	OsWRKY72								
PheWRKY73-1	D		640	0.00E+00	OsWRKY73				OsWRKY73			BdWRKY70	D
PheWRKY73-2	D	D	625	0.00E+00	OsWRKY73	AtWRKY72		U					
PheWRKY74-1	D	D	407	1.00E-116	OsWRKY74				OsWRKY74	D			
PheWRKY74-2	D	U	352	1.00E-100	OsWRKY74								
PheWRKY75	D		339	5.00E-96	OsWRKY75								
PheWRKY76	U	U	398	1.00E-114	OsWRKY76				OsWRKY76			BdWRKY14	
PheWRKY77-1			197	1.00E-53	OsWRKY77				OsWRKY77			BdWRKY52	D
PheWRKY77-2			165	1.00E-43	OsWRKY77								
PheWRKY78		U	1039	0.00E+00	OsWRKY78	AtWRKY20			OsWRKY78	D		BdWRKY64	
PheWRKY79-2			214	2.00E-58	OsWRKY79								

PheWRKY79-1			201	2.00E-54	OsWRKY79							
PheWRKY80-1			887	0.00E+00	OsWRKY80				OsWRKY80			
PheWRKY80-2			556	1.00E-161	OsWRKY80							
PheWRKY80-3	D	D	471	1.00E-135	OsWRKY80							
PheWRKY82			676	0.00E+00	OsWRKY82	AtWRKY4	D	U	OsWRKY82		BdWRKY38	
PheWRKY83-1			552	1.00E-160	OsWRKY83				OsWRKY83	U	BdWRKY63	
PheWRKY83-2			442	1.00E-127	OsWRKY83							
PheWRKY83-3	D		521	1.00E-151	OsWRKY83	AtWRKY21					BdWRKY31	D
PheWRKY85-1		D	665	0.00E+00	OsWRKY85				OsWRKY85	U	BdWRKY61	
PheWRKY85-2			658	0.00E+00	OsWRKY85							
PheWRKY88-1	U	U	376	1.00E-107	OsWRKY88				OsWRKY88		BdWRKY73	
PheWRKY88-2			252	9.00E-77	OsWRKY88							
PheWRKY89	D	D	155	6.00E-41	OsWRKY89				OsWRKY89			
PheWRKY93			276	2.00E-77	OsWRKY93				OsWRKY93			
PheWRKY95			227	4.00E-27	OsWRKY95							
PheWRKY96-1	U	U	415	1.00E-118	OsWRKY96				OsWRKY96		BdWRKY67	
PheWRKY96-2			387	1.00E-110	OsWRKY96							
PheWRKY96-3	U	U	323	5.00E-91	OsWRKY96							
PheWRKY97-1	D	D	616	1.00E-179	OsWRKY97				OsWRKY97			
PheWRKY97-2	U		500	1.00E-144	OsWRKY97						BdWRKY48	
PheWRKY100			332	7.00E-94	OsWRKY100				OsWRKY100			
PheWRK109			248	9.00E-69	OsWRKY86						BdWRKY8	
PheWRKY111	U		315	6.00E-89	OsWRKY12							
PheWRKY114-1	U		264	2.00E-73	OsWRKY52							
PheWRKY114-2			238	1.00E-65	OsWRKY52				OsWRKY52			
PheWRKY116			303	2.00E-85	OsWRKY18				OsWRKY18		BdWRKY85	
PheWRKY119			145	1.00E-37	OsWRKY17						BdWRKY57	
PheWRKY125	D		801	0.00E+00	OsWRKY61							

Note: ‘D’ indicates that the corresponding gene was significantly down-regulated under abiotic stress treatment. ‘U’ indicates up-regulation under abiotic stress treatment.

Supplementary Table S3: The characteristics of *PheWRKY* genes.

Name	ID	NCBI accession No.	Protein size/aa	Moecluar Weight/D	Isoelectric point
PheWRKY1-1	PH01000048G1770		561	58407.3	8.7
PheWRKY1-2	PH01003108G0020		440	46636.8	9.3
PheWRKY2	TCONS_00100293		313	34044.6	7.5
PheWRKY3-1	PH01000716G0640		293	31718.2	6.7
PheWRKY3-2	PH01003110G0050		301	34580.6	6.7
PheWRKY4	PH01000716G0820		412	44251.9	6.5
PheWRKY5-1	PH01000445G0180		460	48622.4	6.8
PheWRKY5-2	PH01000212G0380		291	31392	7.6
PheWRKY7	PH01000271G0420	FP099367	220	23128.4	7
PheWRKY8	PH01001601G0220		333	35824.5	5.7
PheWRKY9-1	PH01000326G0720		525	54931	9.7
PheWRKY9-2	PH01001671G0400		346	35926.3	11.5
PheWRKY10	TCONS_00125853		174	19128	7.4
PheWRKY11-1	PH01000121G1380		339	36899	6.6
PheWRKY11-2	PH01000256G0030		340	37290.5	6.3
PheWRKY11-3	PH01003579G0140		131	14320	8.7
PheWRKY12	PH01000256G0230		325	35075.1	5.1
PheWRKY13-1	PH01000018G0370	FP094710	288	31384	5.4
PheWRKY13-2	PH01000746G0560	FP095416	290	31536	5.4
PheWRKY13-3	PH01001953G0270	FP096670	260	29154.5	4.7
PheWRKY14	PH01000814G0820		333	36236.5	8.5
PheWRKY15-1	PH01000182G0790		309	32441.8	8.5
PheWRKY15-2	PH01021666G0010		223	23684.2	6.3
PheWRKY16	PH01004514G0080	FP093080	357	37576.9	7.8
PheWRKY17-1	PH01000622G0760		563	62243.5	6.3
PheWRKY17-2	PH01002711G0120		555	61131.4	6.5
PheWRKY17-3	PH01000478G0060		159	17277.1	4.7
PheWRKY19-1	PH01000046G1680		288	31265.3	6.3
PheWRKY19-2	PH01000242G1360	FP091320	264	29034.7	6.2
PheWRKY19-3	PH01002396G0160	FP097832	310	34149.5	5.6
PheWRKY21	PH01001280G0060	FP093287	273	29681.7	8

PheWRKY22-1	PH01000009G1940		269	30206.8	6.9
PheWRKY22-2	PH01000112G1110		343	38421.7	9
PheWRKY24-1	PH01001737G0080		568	60581	6.5
PheWRKY24-2	PH01002744G0230		571	61038.8	6.4
PheWRKY25	PH01003558G0150		106	11037	5.6
PheWRKY26-1	PH01002011G0380		193	20781.9	6.2
PheWRKY26-2	PH01001210G0270		200	21525.8	8.5
PheWRKY28	PH01001777G0070		272	28886.6	9.4
PheWRKY29-1	PH01002776G0100		302	31619.2	5.9
PheWRKY29-2	PH01000549G0590		301	31612.3	6.3
PheWRKY29-3	PH01001331G0040	FP098810	262	28392.3	9.8
PheWRKY29-4	PH01001331G0070		246	25611.5	8.4
PheWRKY34-1	TCONS_00062338		228	25302.6	8.1
PheWRKY34-2	PH01000218G0100	FP098149	228	25596	8.5
PheWRKY35-1	PH01000035G0560		762	81687.6	6.5
PheWRKY35-2	PH01001142G0610		568	61358	5.5
PheWRKY36	TCONS_00005299		234	25783.6	8.6
PheWRKY39-1	PH01001232G0560		358	38382.9	5.3
PheWRKY39-2	PH01029174G0010		253	26479.3	5.8
PheWRKY39-3	PH01002558G0060		244	25935	7.1
PheWRKY42	PH01001515G0160	FP094685	263	28155.5	9.7
PheWRKY43	PH01000242G0250		561	60003.7	6.4
PheWRKY44	PH01001698G0040		310	32158.5	6.4
PheWRKY45-1	PH01002378G0050		301	31982.4	5.9
PheWRKY45-2	PH01003908G0140		163	17880.2	4.5
PheWRKY46-1	PH01000130G0620		349	38718.6	9.5
PheWRKY46-2	PH01000524G0850	FP099236	223	25178.7	9.1
PheWRKY48-1	PH01000659G0050	FP098977	302	32508.8	4.7
PheWRKY48-2	PH01002238G0110		341	36843.2	5.5
PheWRKY49	PH01000298G0170	FP101665	378	40592.3	6.6
PheWRKY51-2	PH01000557G0340		215	23770.6	9.8
PheWRKY51-1	PH01002220G0280		321	33944.4	9.6
PheWRKY65-1	PH01000130G0430		340	36540.5	5.4
PheWRKY65-2	PH01001070G0190		274	29426.5	5.9

PheWRKY53-1	PH01000296G0390	FP094324	490	51666.9	8.1
PheWRKY53-2	PH01004067G0040		368	38931.9	8.4
PheWRKY55	PH01000430G0510		210	23655.3	8.5
PheWRKY62	PH01000534G0230		181	19971.5	6.7
PheWRKY66	PH01002035G0390		489	51193	5.1
PheWRKY67-1	PH01001131G0560	FP095669	190	20659.9	6.6
PheWRKY67-2	PH01004940G0100	FP098478	192	20827	5.8
PheWRKY68-1	PH01000224G1000		297	31387	10
PheWRKY68-2	PH01278028G0010		92	10214.9	11
PheWRKY69-1	PH01002800G0110	FP099440	313	33753.6	6.2
PheWRKY69-2	PH01002018G0330	FP098490	317	33996.1	6.3
PheWRKY70-1	PH01000206G0250	FP098651	560	59322.3	5.8
PheWRKY70-2	PH01001037G0370		505	54107.4	6.1
PheWRKY71-1	PH01000735G0110	FP092380	338	36542.2	6.5
PheWRKY71-2	PH01000043G1290		339	36997.8	8.6
PheWRKY72-1	PH01001100G0250		209	22804.9	9.1
PheWRKY72-2	TCONS_00109438		206	22633.6	9.5
PheWRKY72-3	PH01005961G0010	FP101056	176	20364.6	9.1
PheWRKY73-1	PH01005137G0020		594	62870.1	6.8
PheWRKY73-2	PH01002537G0070		604	64021.4	7.2
PheWRKY74-1	PH01003922G0080		341	36882.3	5.8
PheWRKY74-2	PH01001397G0220	FP097435	290	31237	7.2
PheWRKY75	PH01001883G0330		324	34339.2	5.4
PheWRKY76	PH01000405G0510		302	32178.9	8.4
PheWRKY77-1	PH01001004G0240		242	25617.3	6.3
PheWRKY77-2	PH01001362G0060		240	26174.2	8.6
PheWRKY78	PH01001245G0180		591	63226.2	5.9
PheWRKY79-2	PH01001722G0210		192	21302.3	8.3
PheWRKY79-1	PH01002142G0270		272	28116.4	10.8
PheWRKY80-1	PH01000050G1350		710	76317.7	6
PheWRKY80-2	PH01001626G0320		699	75338.4	5.8
PheWRKY80-3	PH01003485G0070		921	100477.4	7.7
PheWRKY82	PH01003170G0110		511	56643.3	5.9
PheWRKY83-1	PH01000025G1260		347	37475.5	10.1

PheWRKY83-2	PH01001154G0200		358	38331.5	10.9
PheWRKY83-3	PH01001697G0180		355	38671.9	9.9
PheWRKY85-1	PH01000006G0810		410	43314	6.5
PheWRKY85-2	PH01000463G0800		363	39999.6	8
PheWRKY88-1	PH01000535G0790		369	38535.4	6.1
PheWRKY88-2	PH01000371G0960		322	34656.3	5.9
PheWRKY89	PH01000823G0340		204	22209.9	6.5
PheWRKY93	PH01000216G0630		219	24330.7	10.3
PheWRKY95	PH01000986G0410		341	36471.7	7
PheWRKY96-1	PH01001978G0130		386	41870.8	5.2
PheWRKY96-2	PH01002022G0200		357	39524.6	6.6
PheWRKY96-3	PH01001458G0040		406	44721	6.4
PheWRKY97-1	PH01000906G0240		549	59019.9	6.4
PheWRKY97-2	PH01000274G0230	FP098043	484	50820	8.4
PheWRKY100	PH01001070G0240		285	32530	6.9
PheWRK109	PH01000009G2100		296	32119.8	6.7
PheWRKY111	PH01003057G0080		299	32557.5	5.9
PheWRKY114-1	PH01001070G0420	FP093249	329	36178.5	6.2
PheWRKY114-2	PH01002396G0190		343	37227.4	5.9
PheWRKY116	PH01000009G1960		309	34600.6	6.6
PheWRKY119	PH01004450G0140		325	34518.9	6.3
PheWRKY125	PH01001500G0110		1168	131060	7.3

Supplementary Table S4. Variants of the heptapeptide WRKYGQK in four species.

Variants	Protein sequences	Groups
WRKYGKK	PheWRKY7	group2c
WRKYGKK	PheWRKY26-1	group2c
WRKYGKK	PheWRKY26-2	group2c
WKKYGQK	PheWRKY11-3	group2c
WRKYGEK	PheWRKY46-1	group3
WRKYGEK	PheWRKY46-2	group3
WRKYGEK	PheWRKY114-1	group3
WRKYGEK	PheWRKY114-2	group3
WRKYGEK	PheWRKY55	group3
WRKKFG	PheWRKY125C	group1
WRKYGKK	PheWRKY67-1	group2c
WRKYGKK	PheWRKY67-2	group2c
WRKYGKK	PheWRKY74-2	group3
CRKYGQA	PheWRKY80-1N	group1
WRKYGQQ	PheWRKY80-2N	group1
WRKYGKK	OsWRKY7	group2c
WRKYGKK	OsWRKY10	group2c
WRKYGKK	OsWRKY77	group2c
WRKYGKK	OsWRKY67	group2c
WRKYGKK	OsWRKY26	group2c
WKYGEK	OsWRKY46a	group3
WKYGEK	OsWRKY46b	group3
WKYGEK	OsWRKY55	group3
WKYGEK	OsWRKY91	group3
WKYGEK	OsWRKY52	group3
WKYGEK	OsWRKY81	group3
WRKYGKK	BdWRKY56	group2c
WRKYGKK	BdWRKY50	group2c
WRKYGKK	BdWRKY58	group2c
WRKYGKK	BdWRKY2	group2c
WRKYGKK	BdWRKY52	group2c
WRKYGQT	BdWRKY62N	group1
WRKYGQT	BdWRKY62C	group1
WRKKGPK	BdWRKY60N	group1
WRKYGEK	BdWRKY43	group3
WRKYGEK	BdWRKY44	group3
WRKYGEK	BdWRKY78	group3
WRKYGKK	AtWRKY50	group2c
WRKYGKK	AtWRKY51	group2c
WRKYGKK	AtWRKY59	group2c

Supplementary Table S5. Estimated divergence period of WRKY gene pairs in four species.

Paralogous pairs	Ks	Ka	ka/ks	Data (million years ago)
PheWRKY1-1/PheWRKY1-2	0.0935	0.0388	0.414973	7.19
PheWRKY3-1/PheWRKY3-2	0.1337	0.0485	0.362752	10.28
PheWRKY11-1/PheWRKY11-2	0.121	0.0391	0.32314	9.31
PheWRKY13-1/PheWRKY13-2	0.1747	0.056	0.32055	13.44
PheWRKY15-1/PheWRKY15-2	0.0914	0.0511	0.559081	7.03
PheWRKY17-1/PheWRKY119	1.278	0.9078	0.710329	98.31
PheWRKY19-1/PheWRKY19-2	0.1362	0.1096	0.804699	10.48
PheWRKY22-1/PheWRKY22-2	0.2146	0.0743	0.346226	16.51
PheWRKY24-1/PheWRKY24-2	0.1754	0.0439	0.250285	13.49
PheWRKY26-1/PheWRKY26-2	0.1793	0.0308	0.171779	13.79
PheWRKY29-1/PheWRKY29-2	0.0862	0.0374	0.433875	6.63
PheWRKY29-3/PheWRKY29-4	0.1934	0.1133	0.585832	14.88
PheWRKY34-2/PheWRKY36	0.5136	0.1541	0.300039	39.51
PheWRKY35-1/PheWRKY35-2	0.1877	0.0826	0.440064	14.44
PheWRKY39-1/PheWRKY39-2	0.1768	0.0551	0.311652	13.6
PheWRKY46-1/PheWRKY46-2	0.2999	0.1234	0.41147	23.07
PheWRKY48-1/PheWRK48-2	0.1406	0.0754	0.536273	10.82
PheWRKY51-1/PheWRKY51-2	0.2191	0.0502	0.229119	16.85
PheWRKY65-1/PheWRKY65-2	0.3104	0.1396	0.449742	23.88
PheWRKY53-1/PheWRKY53-2	0.0943	0.1254	1.329799	7.25
PheWRKY67-1/PheWRKY67-2	0.085	0.0644	0.757647	6.54
PheWRKY69-1/PheWRKY69-2	0.1234	0.0646	0.523501	9.49
PheWRKY70-1/PheWRKY70-2	0.1194	0.0456	0.38191	9.18
PheWRKY71-1/PheWRKY71-2	0.2114	0.0536	0.253548	16.26
PheWRKY72-1/PheWRKY72-2	0.5873	0.1786	0.304104	45.18
PheWRKY73-1/PheWRKY73-2	0.1314	0.0591	0.449772	10.11
PheWRKY74-1/PheWRKY74-2	0.1841	0.0551	0.299294	14.16
PheWRKY77-1/PheWRKY77-2	0.2112	0.1125	0.53267	16.25
PheWRKY80-1/PheWRKY80-2	0.1455	0.0488	0.335395	11.19
PheWRKY83-1/PheWRKY83-3	0.8084	0.1348	0.166749	62.18
PheWRKY96-1/PheWRKY96-2	0.1807	0.1611	0.891533	13.9
PheWRKY97-1/PheWRKY97-2	0.2524	0.1592	0.630745	19.42
PheWRKY114-1/PheWRKY114-2	0.2064	0.1282	0.621124	15.88
PheWRKY12/PheWRKY111	0.5551	0.2245	0.404432	42.7
PheWRKY42/PheWRKY66	0.5378	0.6752	1.255485	41.37
PheWRKY21/PheWRKY95	1.1343	0.8645	0.762144	87.25
BdWRKY7/BdWRKY47	0.242	0.0895	0.369835	18.61
BdWRKY36/BdWRKY42	0.3022	0.4294	1.420913	23.25
BdWRKY15/BdWRKY23	0.9037	0.7672	0.848954	69.51
BdWRKY1/BdWRK5	0.5602	0.6147	1.097287	43.09
BdWRKY6/BdWRKY35	0.3006	0.3445	1.146041	23.12
BdWRKY7/BdWRKY47	0.242	0.0895	0.369835	18.62
BdWRKY11/BdWRKY79	0.6226	0.5248	0.842917	47.89
BdWRKY16/BdWRKY76	0.5975	0.4596	0.769205	45.96

BdWRKY17/BdWRKY68	0.7927	0.906	1.142929	60.98
BdWRKY18/BdWRKY59	0.1069	0.0977	0.913938	8.22
BdWRKY19/BdWRKY40	0.4991	0.5391	1.080144	38.39
BdWRKY23/BdWRKY71	0.6683	0.3603	0.539129	51.41
BdWRKY24/BdWRKY54	0.6332	0.3134	0.494946	48.71
BdWRKY25/BdWRKY83	0.726	0.7788	1.072727	55.85
BdWRKY31/BdWRKY63	1.0966	0.2511	0.22898	84.35
BdWRKY33/BdWRKY41	0.5569	0.2569	0.461304	42.84
BdWRKY36/BdWRKY42	0.3022	0.4294	1.420913	23.25
BdWRKY39/BdWRKY68	0.3677	0.3805	1.034811	28.28
BdWRKY43/BdWRKY78	0.5152	0.5528	1.072981	39.63
BdWRKY45/BdWRKY46	0.322	0.3774	1.17205	24.77
BdWRKY48/BdWRKY70	0.9179	0.5415	0.589934	70.61
BdWRKY51/BdWRKY55	0.5164	0.398	0.77072	39.72
BdWRKY61/BdWRKY66	0.8179	0.489	0.597873	62.92
BdWRKY81/BdWRKY82	0.5629	0.4629	0.822349	43.3
OsWRKY40/OsWRKY64	0.0749	0.0401	0.535381	5.76
OsWRKY41/OsWRKY61	0.5329	0.5723	1.073935	40.99
OsWRKY46a/OsWRKY46b	0.0402	0.0136	0.338308	3.09
OsWRKY50/OsWRKY65	0.0827	0.0584	0.706167	6.36
OsWRKY56/OsWRKY100	0.2146	0.1206	0.561976	16.51
OsWRKY52/OsWRKY81	0.1855	0.0504	0.271698	14.27
OsWRKY1/OsWRKY53	0.5291	0.4193	0.792478	40.7
OsWRKY2/OsWRKY88	1.1514	0.4675	0.406027	88.57
OsWRKY3/OsWRKY29	0.4272	0.3265	0.764279	32.86
OsWRKY5/OsWRKY9	0.3896	0.382	0.980493	29.97
OsWRKY8/OsWRKY11	0.6375	0.4731	0.742118	49.04
OsWRKY10/OsWRKY67	0.3778	0.4527	1.198253	29.06
OsWRKY12/OsWRKY111	0.7158	0.2976	0.415759	55.06
OsWRKY13/OsWRKY14	0.5645	0.452	0.800709	43.42
OsWRKY15/OsWRKY19	0.516	0.5085	0.985465	39.69
OsWRKY16/OsWRKY49	0.4125	0.4431	1.074182	31.73
OsWRKY18/OsWRKY116	0.6762	0.3951	0.584295	52.02
OsWRKY24/OsWRKY70	0.4426	0.2788	0.629914	34.05
OsWRKY25/OsWRKY68	0.1937	0.3223	1.663913	14.9
OsWRKY28/OsWRKY71	0.2475	0.2697	1.089697	19.03
OsWRKY30/OsWRKY80	0.8966	0.3142	0.350435	68.97
OsWRKY31/OsWRKY39	0.5149	0.5218	1.013401	39.61
OsWRKY36/OsWRKY79	0.6362	0.4306	0.676831	48.94
OsWRKY37/OsWRKY66	0.4515	0.3973	0.879956	34.73
OsWRKY48/OsWRKY54	0.553	0.4969	0.898553	42.54
OsWRKY62/OsWRKY76	0.4974	0.5117	1.028749	38.26
OsWRKY69/OsWRKY74	0.5345	0.4531	0.847708	41.12
OsWRKY73/OsWRKY97	0.5342	0.4898	0.916885	41.09
OsWRKY81/OsWRKY114	1.3251	0.8598	0.648857	101.9
OsWRKY86/OsWRKY109	1.1318	0.9705	0.857484	87.06
OsWRKY95/OsWRKY115	1.27915	0.8318	0.650276	98.41
OsWRKY87/OsWRKY122	1.1464	1.0066	0.878053	88.18
AtWRKY41/AtWRKY53	0.392	0.381	0.973194	13.06

AtWRKY54/AtWRKY70	0.939	0.411	0.437899	31.29
AtWRKY63/AtWRKY64	0.397	0.138	0.346862	13.22
AtWRKY2/AtWRKY34	0.606	0.325	0.53529	20.21
AtWRKY3/AtWRKY4	0.782	0.141	0.180598	26.08
AtWRKY7/AtWRKY15	1.617	0.454	0.280475	53.9
AtWRKY8/AtWRKY28	0.368	0.431	1.170699	12.26
AtWRKY11/AtWRKY17	0.844	0.151	0.178952	28.13
AtWRKY14/AtWRKY35	0.656	0.28	0.427025	21.86
AtWRKY18/AtWRKY60	0.847	0.215	0.253511	28.24
AtWRKY22/AtWRKY27	1.352	0.587	0.43395	45.07
AtWRKY24/AtWRKY56	0.547	0.158	0.288564	18.22
AtWRKY31/AtWRKY6	0.454	0.464	1.021375	15.13
AtWRKY39/AtWRKY74	0.861	0.115	0.133132	28.69
AtWRKY65/AtWRKY69	0.697	0.585	0.838927	23.24
AtWRKY12/AtWRKY13	1.1728	0.4989	0.425392	14.18
AtWRKY45/AtWRKY75	0.3862	0.4399	1.139047	37.97
AtWRKY51/AtWRKY50	0.6745	0.7752	1.149296	38.31
AtWRKY61/AtWRKY72	0.7979	0.6367	0.79797	26.6

Ks: synonymous substitution rate; *Ka*: non-synonymous substitution rate; MYA: million years ago.

Supplementary Table S6. The fold change of up-regulated genes in various abiotic stresses and physiological processes.

Cold stress							
	1h	1h	24h		6h	12h	24h
PheWRKY1-1	3.77	**50.67**	21.31	PheWRKY49	1.69	3.79	**8.05**
PheWRKY4	1.09	**5.01**	**61.88**	PheWRKY65-1	9.04	2.66	**9.76**
PheWRKY5-1	0.42	**4.20**	**60.22**	PheWRKY114-1	**5.53**	0.58	0.81
PheWRKY26-2	**4.70**	**7.78**	**44.60**	PheWRKY55	**4.85**	0.32	**11.39**
PheWRKY8	3.75	1.47	**6.13**	PheWRKY62	1.70	**13.44**	**45.31**
PheWRKY11-2	**21.20**	2.25	**9.61**	PheWRKY69-1	3.79	**14.66**	**18.81**
PheWRKY111	**7.44**	0.45	0.49	PheWRKY69-2	**16.26**	3.02	2.27
PheWRKY13-2	0.56	2.02	**4.26**	PheWRKY70-2	**7.16**	0.27	0.85
PheWRKY19-1	**46.01**	**5.79**	**53.69**	PheWRKY72-1	1.15	0.94	**6.20**
PheWRKY19-3	1.62	**9.18**	3.06	PheWRKY72-3	3.44	**4.40**	2.41
PheWRKY24-2	2.28	0.51	**4.10**	PheWRKY76	**17.27**	2.14	2.36
PheWRKY36-1	**31.51**	1.46	1.82	PheWRKY78	1.08	2.39	**5.23**
PheWRKY45-1	**5.43**	0.89	2.12	PheWRKY88-1	1.97	1.28	**12.35**
PheWRKY48-2	1.96	**5.19**	**8.68**	PheWRKY96-1	1.96	**5.19**	**8.98**
Drought stress							
	1h	12h	24h		1h	12h	24h
PheWRKY1-1	2.08	**16.82**	**13.35**	PheWRKY65-1	**8.91**	1.96	**31.88**
PheWRKY4	0.53	3.98	**11.24**	PheWRKY55	3.00	0.86	**12.35**
PheWRKY5-1	1.85	**4.52**	**18.14**	PheWRKY62	**4.28**	0.81	3.29
PheWRKY26-2	**5.92**	0.88	**10.69**	PheWRKY69-1	**25.18**	**19.81**	**7.15**
PheWRKY11-2	2.03	0.58	**6.26**	PheWRKY69-2	3.07	**8.39**	**4.27**
PheWRKY15-2	1.37	1.28	**6.29**	PheWRKY72-3	2.16	0.86	**4.59**

PheWRKY19-2	0.18	0.99	**4.04**	PheWRKY71-2	2.74	1.62	**4.57**
PheWRKY34-2	**23.38**	**24.74**	**10.89**	PheWRKY74-2	1.97	**5.84**	1.33
PheWRKY44	0.42	0.40	**4.06**	PheWRKY76	**30.12**	**12.20**	**28.77**
PheWRKY45-1	**8.13**	3.14	3.25	PheWRKY88-1	**6.82**	1.72	1.20
PheWRKY49	1.85	3.28	**5.90**	PheWRKY96-1	**47.62**	1.05	2.06

	Floral stages				Shoot growth	
	F1	F2	F3	F4		
PheWRKY14	1.93	**20.51**	**8.08**	**12.45**	PheWRKY111	**119.71**
PheWRKY17-1	**7.86**	**9.19**	**7.35**	**20.89**	PheWRKY29-3	**13.9**
PheWRKY17-2	**5.24**	1.86	1.15	1.48	PheWRKY45-2	**11.46**
PheWRKY17-3	**5.79**	2.40	1.73	0.03	PheWRKY49	**5.64**
PheWRKY19-1	**8.60**	**4.47**	**3.17**	**5.27**	PheWRKY62	**5.54**
PheWRKY19-2	**7.62**	**4.56**	**5.27**	**20.02**	PheWRKY28	**8.84**
PheWRKY22-1	3.38	**6.53**	**5.05**	3.63	PheWRKY80-3	**5.03**
PheWRKY29-3	2.08	**4.03**	**3.15**	**7.97**		
PheWRKY35-1	**4.24**	**4.96**	**9.38**	0.35		
PheWRKY35-2	**63.69**	**141.69**	**543.08**	0.02		
PheWRKY34-2	**16.29**	**14.68**	2.65	0.39		
PheWRKY39-1	2.41	2.87	1.68	**9.42**		
PheWRKY39-2	1.37	1.45	1.29	**5.21**		
PheWRKY39-3	**28.95**	**5.59**	0.21	**31.12**		
PheWRKY43	0.20	**6.33**	**178.12**	**4.22**		
PheWRKY48-1	**10.94**	**5.31**	**10.88**	**15.40**		
PheWRKY48-2	**7.03**	3.60	2.60	**14.22**		
PheWRKY49	2.34	2.84	3.07	**7.60**		
PheWRKY65-1	**6.37**	**4.99**	**5.14**	**19.25**		
PheWRKY114-1	0.91	2.36	0.96	**5.24**		
PheWRKY62	0.92	1.87	0.81	**7.08**		
PheWRKY67-2	2.16	2.24	1.45	**4.13**		
PheWRKY74-1	**4.10**	1.46	**3.04**	**5.69**		
PheWRKY79-1	**22.72**	**15.49**	**4.89**	3.35		
PheWRKY109	1.15	0.45	1.35	**6.86**		
PheWRKY96-1	**5.38**	1.75	0.90	1.09		
PheWRKY97-1	0.64	1.24	**4.94**	**29.50**		

Note: The up-regulated time points are shown in bold.

Supplementary Table S7. The primer sequences used for SqRT-PCR and qRT-PCR.

Gene name	Forward primer	Reverse primer	Amplification efficiency
PheWRKY1-1	GTCGAAGTTCTCCGGATTGC	CGCCTATGATGGACGTGATG	0.95
PheWRKY3-2	CGTTCATGACCAAGAGCGAG	GGAGGGGTCGTCTGAAGATC	0.92
PheWRKY4	GGGGTGGTTTTGATCATGGG	GTGGAGGTATGACGGGTAGG	0.88
PheWRKY5-1	CGACGGTGGAAGGACTAACT	ACTTCCTCCATTGGCATCCA	1.07
PheWRKY7	GTCCTCCTACTTCTCCCACG	CTAGCGTGAGTGGTTGCAC	1.03
PheWRKY8	GAGGAGACCATGGCCTCTAC	AGTCGGTGATGCTGGAGTAC	0.97
PheWRKY9-1	GGTCCGAAGCACCAATGATC	CCTTCTTCATCTTGCTGGCC	1.13
PheWRKY11-2	CTTCTTCAATCAGCTGGCGG	ACTCATCACATCGACCGACA	0.93
PheWRKY12	CTTCAACGATTGGGACCTGC	GGTACTCCAAGTCGTACAGC	0.98
PheWRKY13-1	GGAACAGGAGAAGGAGCAGA	CTCGCGATCCCACTCCTC	0.97

PheWRKY13-2	GCCGAGGAAGAGGAAAGCTT	CAAACAGCGCTTCCTCCC	1.02
PheWRKY13-3	TGCTCGTCACCTACTCGTAC	GGGTCTCTGGCGTATCTACC	0.96
PheWRKY14	CCGGGGATGAAGGAGGATTT	TCCAATAGATCCTGCGTGCA	0.98
PheWRKY15-1	ACGTCAGAGTGCCAGGTTAG	GCCCGAGTTACCCAAGAAA	1.02
PheWRKY15-2	AGTCAGAGTGCCAGGTTAGC	ATCCAAGAAATCGGCGTCCA	1.12
PheWRKY16	TCGTCAAGAGGGAGATGGTG	GCTCTTGTCACCCTCCTCTT	0.97
PheWRKY19-1	CTCAGCTGGAGGAAGTACGG	CAGGTGTGCTCTCCGATGTA	0.92
PheWRKY19-2	GACGCCCAGTTCAAGAAGAG	GTACACGACGTCGAAGAGCA	0.96
PheWRKY19-3	CTCCTGCTGCAATCCCAATC	TCCATGAGTAGCCATCGTCC	0.96
PheWRKY22-2	TCCCTGCAGCACTAGACAAA	CTTGTAACGTCCTGAAGCCG	0.96
PheWRKY24-1	CTACTTCAACATGCCGACCG	GTCTGGAAGGAGAAGTCGGA	0.95
PheWRKY24-2	CAACTGTTCAACTGGAGGCC	TGTTGCTGCTGACCTCTGTA	1.03
PheWRKY26-2	CACCTCGACGTCTTGGACTA	ATCTCCTCCTCCGACCTCAT	0.97
PheWRKY28	AGGTCTCCAAGCTCTACGTC	GCTGCACCTTCTTCTTGACC	0.92
PheWRKY29-1	ACCCCAGGAGCTACTACAGA	TGAGTACTGTGCATCTCCGG	0.96
PheWRKY29-2	CAACATCAAGTGCACGGTGA	CGGAGGAGGATGGTTTTGGT	1.07
PheWRKY34-2	ATATTGGCGTTGAGGAGGTG	CTTGTAGCCATCATCCAGCA	0.97
PheWRKY39-2	CGTGCCAAGATCCAAGAGAAG	CCGGTGTAGGTGAGGATGAA	0.97
PheWRKY42	CGATTACTCTCACGGGCTCA	GCGTTCTTGGAGCTGATCG	0.94
PheWRKY43	GGATGCCAATGGAGGAAGTA	TCCCCTCGTACGTCGTTATC	0.96
PheWRKY44	ACCGCACAAACAACCTCATC	CTCATCCACCTGCACGAAC	0.98
PheWRKY45-1	GATCCTCCACACCTTCTCCC	AGGTTCTTCATGGTCGTCGT	0.93
PheWRKY46-1	GCAACTCCAACTTCCCAAGG	TGGTACGCGAATTGAAGCAG	0.98
PheWRKY46-2	GAGGAAGTACGGCGAGAAGA	CAGATCGATACGGCCTTCCT	0.98
PheWRKY48-1	CCTCCCTCTACGTCATCACC	TGACTCCACCTTGCACATCT	0.94
PheWRKY48-2	GATCCTCCACACCTTCTCCC	AGGTTCTTCATGGTCGTCGT	1.02
PheWRKY49	AGGGCGAGGAGAGTAAGGAG	GGAAATGGGCTGTTCTTGAC	0.86
PheWRKY51-2	GATGATTACTCGTGGCGCAA	AGGTGACGATGAGCATGGAT	0.92
PheWRKY51-1	GGAGGAGCAGCATATGGACT	GGTAAAGTCCAGCGTCATGG	0.95
PheWRKY53-1	GCCGATCTCATGACCTCTCA	CTTCCTCCAGTTGTACCCGT	0.91
PheWRKY53-2	AAGGAGGATGCTGACAACGA	ACCGTCGTGCACTTGTAGTA	0.97
PheWRKY55	GAACCGATCACCAATGCCAG	TAGAACCGAATCTGCCTGCA	0.94
PheWRKY62	CGAACACAACCATGCTCAAC	TCACAAACTCCGCATCACTC	0.96
PheWRKY65-1	AATTCGAGCGAGCAGGATTA	AACGAGCAATTCATCGATCC	0.95
PheWRKY65-2	TGGCTCATCTACTGCTACCG	CCCGTTGAAGTCCATCTCCT	0.93
PheWRKY66	GATACTGGTGGGGTGTTGGA	GTTCTTCCTGCGCTTGATCC	0.89
PheWRKY67-1	AGGATTGGGTTCAGGACGAG	TAGGTGGTGATGACGTAGCG	1.03
PheWRKY67-2	GGCTTCGTTGGGACTAAACC	CTCTCGCTCTCTTCTCCTCG	1.01
PheWRKY69-1	GTGAACACGGACAGCAACC	ACGTCTGCCACTCTCTGTAC	0.89
PheWRKY69-2	CTCATCGACAGGTCCATCGG	CTCACCTGACTCGTCCACTT	0.92
PheWRKY70-1	CTCACCGATCCTCCTCATCC	GTTGTACGAGTCTCCACCCA	0.91
PheWRKY70-2	GCGGACTTCATGATGCCATT	CTGCACTTGTAGTAGCTGCG	0.94
PheWRKY71-1	TCTCCAAGCTCTACGTCCAC	CGCTGCACCTTCTTCTTGAC	0.95
PheWRKY71-2	CAAGCCCAAGATTCTCGTCG	GTGACCATGCCACTGAACTG	0.91
PheWRKY72-1	TTTGCAACACAGCCATCTTC	TGTGGACTTCTTGCACCATC	0.98
PheWRKY72-3	ATGGGGAGCGGTCAGATATG	TACGTCGTCACCACAACAGA	0.95
PheWRKY73-1	ACTAGGGTTTCTGTGAGGGC	CCTCGTAGGTGGTGATCAGG	0.88
PheWRKY73-2	CCACAAGGGCAGAGATGGAT	TCAGGCTCCTCAATGTTGGT	0.94
PheWRKY74-1	CGTGCCTTTCAAGACCAACA	CATCCCTGAGAATTGCGGTG	0.96

PheWRKY74-2	TCGCAGATCATCTCCCTCAC	TTCCGCTTCTCCATCGTCTT	0.93
PheWRKY75	GTCGACGTCCATCCACCTT	CTGCCGGAGTTGAACATGAC	0.92
PheWRKY76	AAGATCCTGGAGGCCAAGTT	ATTCGACGTCTCCATGCTCT	0.95
PheWRKY77-1	CGATGTCAGCGAGGAGTACT	AGCCCTTGTCGATCTTGTCA	1.08
PheWRKY77-2	GTACACCTCTCTCCTCTCGC	ACCACCACCTCCTGCTAATT	0.95
PheWRKY78	TAGCTCTGAAAATGCCGTGC	GGGCTTGGGATGATTGTGAC	0.97
PheWRKY80-1	CAACTTGTGGGTTTGAGCGA	GGATGAAGCAACACGAGACG	0.94
PheWRKY80-2	GGAAGTGCCTGCTCAATCTG	AGAGTGGTTGGACTAAGGCC	0.9
PheWRKY80-3	CATTCGGCCTCACTAATGCC	TTTGAGGATGAGGTGGTGCT	0.97
PheWRKY82	ATGGAGAAGGTGGAGGCAAA	TGACTCATTGCAAACTGGCC	0.93
PheWRKY83-1	GTCCAGTAGTGCCACTCCAT	GATCCCACTGTTGCTCCTCT	0.94
PheWRKY83-3	TGTCTGCGCATTTCCAGTTC	ACGAAGACTTGCCATCCAGA	0.99
PheWRKY85-1	GGGGTTTTGGAATGTCGCAT	TCAGCAGGTTGAAAGGTTGC	0.94
PheWRKY88-1	GCAGTCCAGATCTCCCAGAA	GATCACCTCTCCTCCACCTG	0.91
PheWRKY89	TGCTGGAGGAAATATGGGCA	TTGGACTGGATCTGTGGCAT	1.16
PheWRKY96-1	CCTCGTCACCTAAGCGTAGT	TTCTTGCATTGACGCCACAA	0.92
PheWRKY96-3	GCAGTCCATTCGCAGTACAG	GTTGTAGCCATCGTCAGCAG	0.97
PheWRKY97-2	GAAGCGCACCTCCAATAGTC	GAGCGACTGGTAGTCCCTGA	0.93
PheWRKY111	ATTGGGATCTTGAGGCGGTC	ACGGCGTAATCAAGAGGGG	0.99
PheWRKY114-1	TGTAGCTGCCGAGGTTGTTA	GGAGGAGAAGATGGGGATCG	0.95
PheWRKY114-2	TGAGGAGAGCTGGCAAAAGA	CTGGATTTGCTTTGTGGCCT	0.92
PheWRKY125	ACCCAAATGCGAGGTCTACA	AGGTGAGTTGAAGATCCCGG	0.96
TIP 41	AAAATCATTGTAGGCCATTGTCG	ACTAAATTAAGCCAGCGGGAGTG	0.97
AtABI4	CCCAACATCAACACAACCATCT	CGGACCACCTTTGCCTTT	1.05
β-tubulin	TGCACCAGCTTGTTGAGAAC	TCACAGCTAGCTTTCGCAGA	0.98
AtABA2	CCCAACATCAACACAACCATCT	CGGACCACCTTTGCCTTT	0.87
AtABI5	CAATAAGAGAGGGATAGCGAACGAG	CGTCCATTGCTGTCTCCTCCA	0.89
AtABI2	GATGGAAGATTCTGTCTCAACGATT	GTTTCTCCTTCACTATCTCCTCCG	0.94
AtABI4	CCCAACATCAACACAACCATCT	CGGACCACCTTTGCCTTT	1.05
AtAREB1	CGCCATTGTCATCAGAAGGG	GACCTTGCAGCTGATTCTCG	0.92

Note: The amplification efficiency of primer pairs which used for qRT-PCR were shown in table. '/' represented primer pairs only used for SqRT-PCR.

Supplementary Figure S1. Alignment of PheWRKY domains. Alignment was performed using ClustalX (version 2.1). The 'Group 1N' or 'Group 1C' indicates the N-terminal WRKY domain or the C-terminal WRKY domain of group 1, respectively.

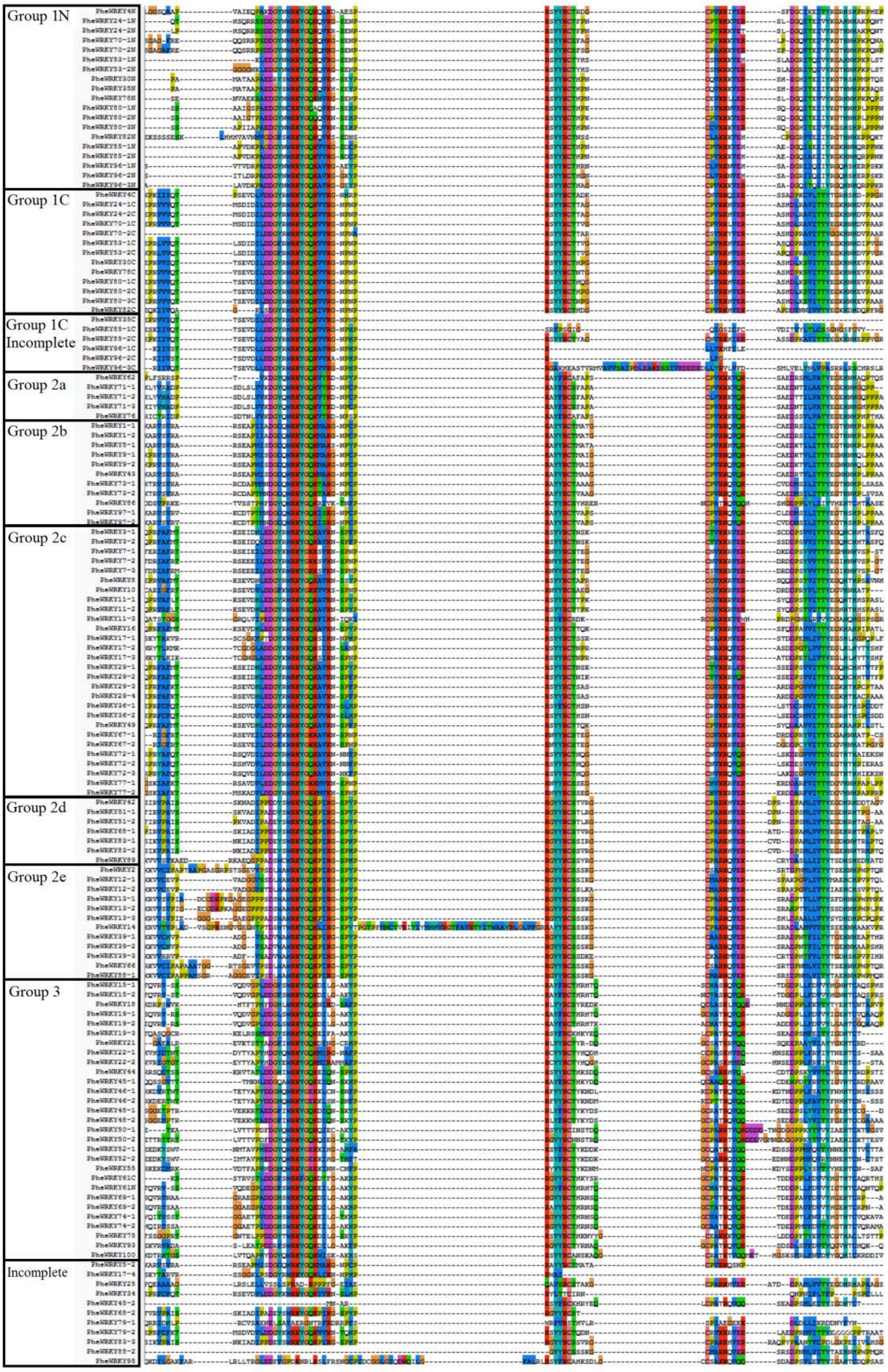

Supplementary Figure S2. Sequence logo of the different motifs identified in the PheWRKY proteins.

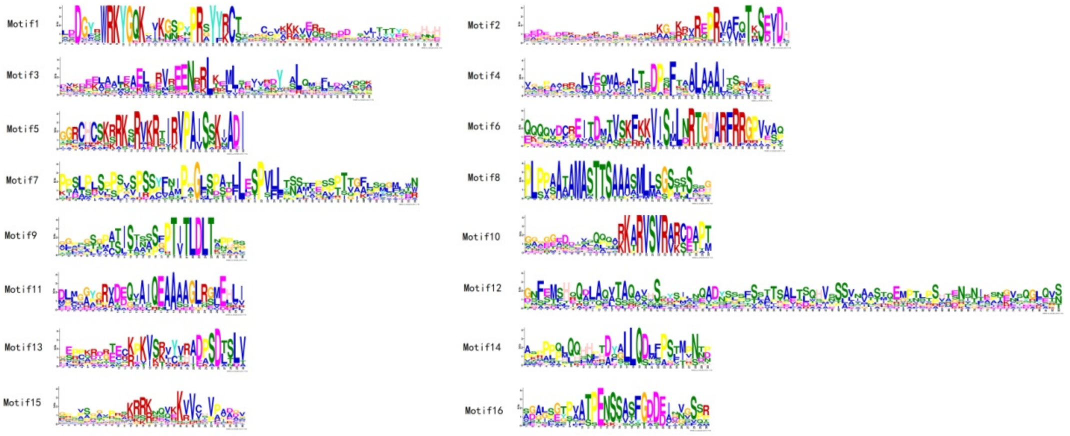

Supplementary Figure S3. Exon/intron structures of the PheWRKY genes

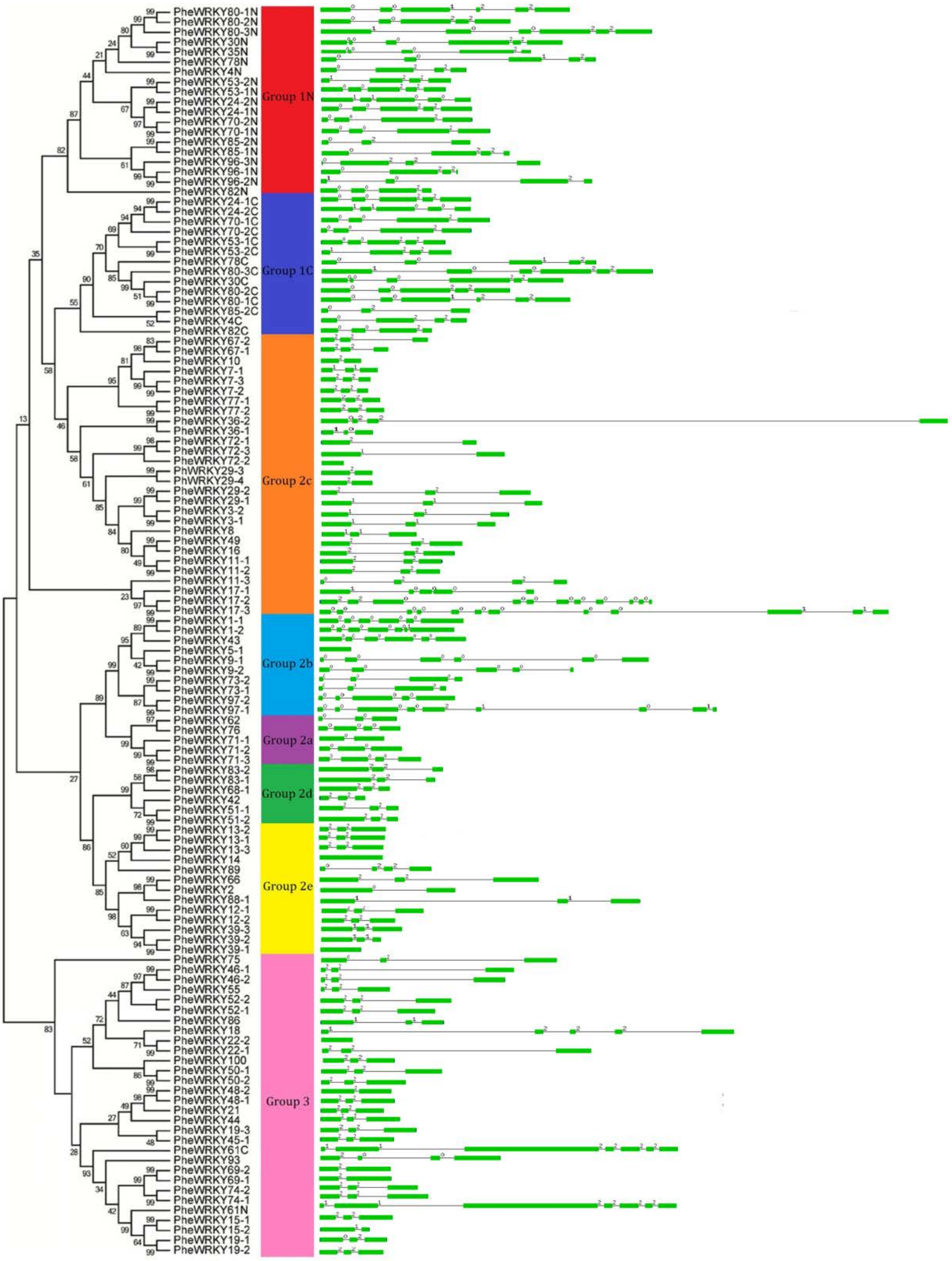

Supplementary Figure S4. Expression profiles of *PheWRKY* genes in various tissues as determined by RT-PCR. R, S, L represented for root, stem and leaf respectively.

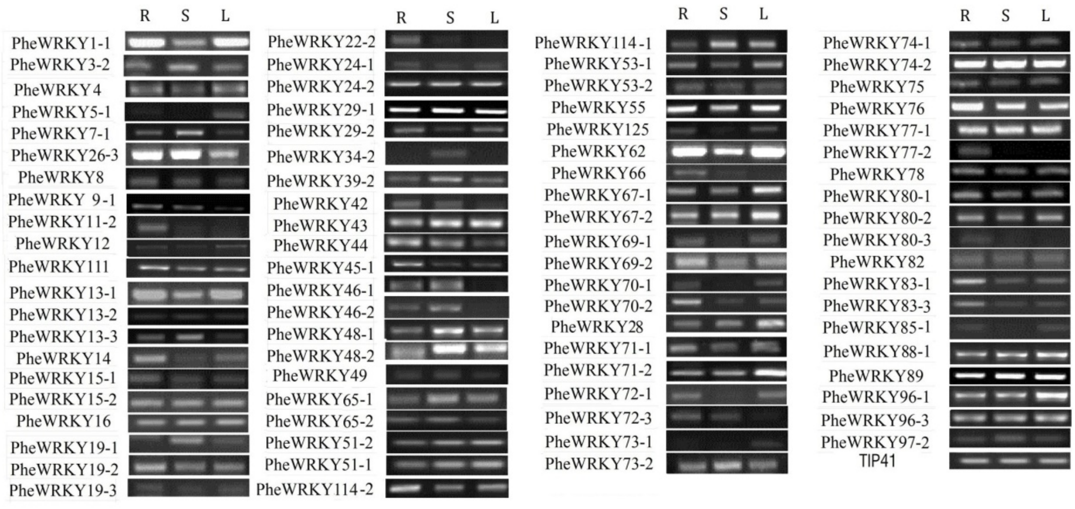

Supplementary Figure S5. Expression verification of four selected WRKY genes by quantitative real-time PCR analysis. Three technical replicates of each biological replicate were repeated. The error bars were computed based on three biological replicates. S, C, F1, F2, F3, F4 and L represent shoot, culm, floral bud formation, inflorescence growing, blooming, embryo formation and leaf, respectively. The expression of each gene in shoot was arbitrarily set at 1.0.

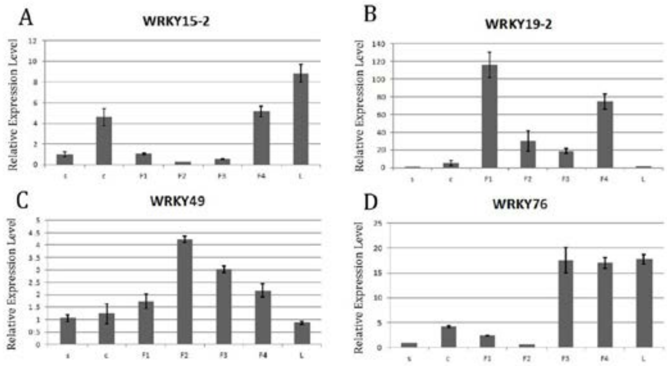

Supplementary Figure S6. The coexpression network of PheWRKYs. Red rectangle represented genes up-regulated in flower development and green rectangle represented genes up-regulated in shoot growth. The network was generated by digital gene expression data and RNA-seq data.

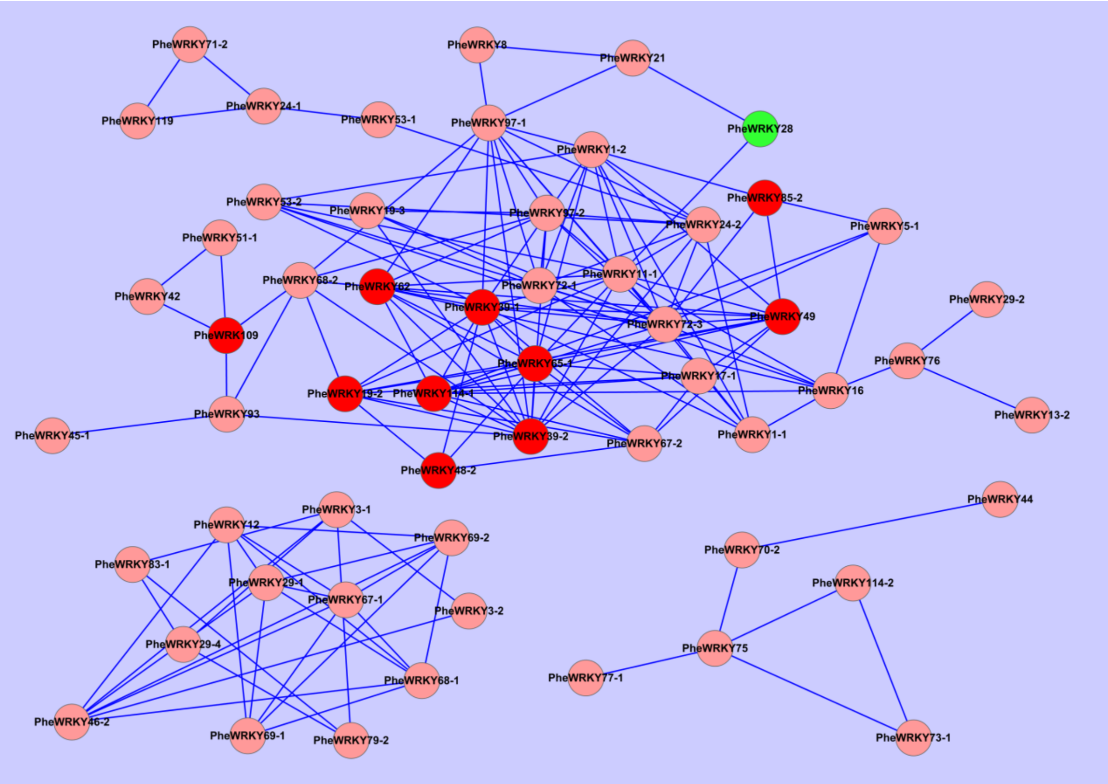

Supplementary Figure S7. Venn diagram showing the classification of genes inducible by cold stress, drought stress, shoot growth, and flower development.

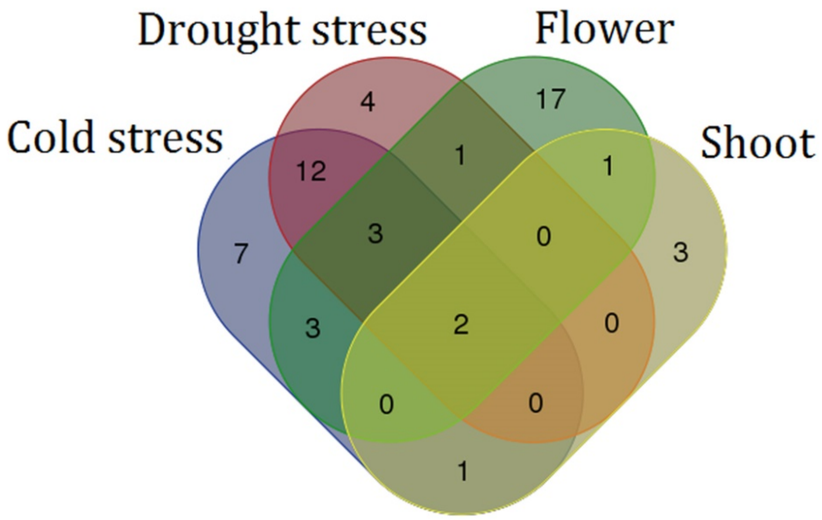

Supplementary Figure S8. Expression patterns of *AtWRKY53* orthologs (*PheWRKY15-1* and *pheWRKY74-1*) in leaf collected from varioug growth period by qPCR. These period including three month old seedlings, three years old young plants and flowering plants.

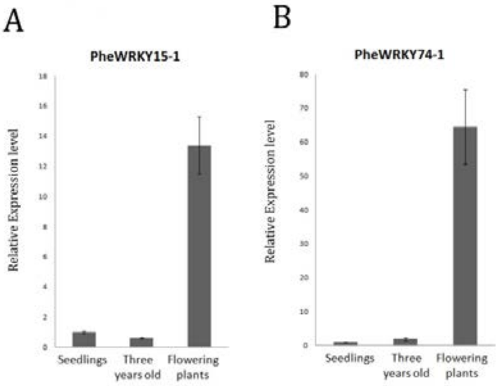
A
PheWRKY15-1
Relative Expression level
18
16
14
12
10
8
6
4
2
0
Seedlings
Three years old
Flowering plants
B
PheWRKY74-1
Relative Expression level
80
70
60
50
40
30
20
10
0
Seedlings
Three years old
Flowering plants
